# Are characiform Fishes Gondwanan in Origin? Insights from a Time-Scaled Molecular Phylogeny of the Citharinoidei (Ostariophysi: Characiformes)

**DOI:** 10.1371/journal.pone.0077269

**Published:** 2013-10-08

**Authors:** Jairo Arroyave, John S. S. Denton, Melanie L. J. Stiassny

**Affiliations:** 1 Department of Ichthyology, Division of Vertebrate Zoology, American Museum of Natural History, New York, New York, United States of America; 2 Department of Biology, the Graduate School and University Center, the City University of New York, New York, New York, United States of America; 3 Richard Gilder Graduate School, American Museum of Natural History, New York, New York, United States of America; Texas A&M University, United States of America

## Abstract

Fishes of the order Characiformes are a diverse and economically important teleost clade whose extant members are found exclusively in African and Neotropical freshwaters. Although their transatlantic distribution has been primarily attributed to the Early Cretaceous fragmentation of western Gondwana, vicariance has not been tested with temporal information beyond that contained in their fragmentary fossil record and a recent time-scaled phylogeny focused on the African family Alestidae. Because members of the suborder Citharinoidei constitute the sister lineage to the entire remaining Afro-Neotropical characiform radiation, we inferred a time-calibrated molecular phylogeny of citharinoids using a popular Bayesian approach to molecular dating in order to assess the adequacy of current vicariance hypotheses and shed light on the early biogeographic history of characiform fishes. Given that the only comprehensive phylogenetic treatment of the Citharinoidei has been a morphology-based analysis published over three decades ago, the present study also provided an opportunity to further investigate citharinoid relationships and update the evolutionary framework that has laid the foundations for the current classification of the group. The inferred chronogram is robust to changes in calibration priors and suggests that the origins of citharinoids date back to the Turonian (*ca* 90 Ma) of the Late Cretaceous. Most modern citharinoid genera, however, appear to have originated and diversified much more recently, mainly during the Miocene. By reconciling molecular-clock- with fossil-based estimates for the origins of the Characiformes, our results provide further support for the hypothesis that attributes the disjunct distribution of the order to the opening of the South Atlantic Ocean. The striking overlap in tempo of diversification and biogeographic patterns between citharinoids and the African-endemic family Alestidae suggests that their evolutionary histories could have been strongly and similarly influenced by Miocene geotectonic events that modified the landscape and produced the drainage pattern of Central Africa seen today.

## Introduction

Vicariance biogeography [1-3] emerged in the late 1970’s as an approach to explain distribution patterns of biotas by linking predictions of phylogenetic systematics [4] and plate tectonics [5,6]. The vicariance model proposes that large-scale plate-tectonic-driven geomorphological processes (e.g., orogenic uplift, continental drift) are sufficient to explain the disjunct distribution of sister lineages [2]. Despite the fact that vicariant scenarios only hold true if cladogenetic events and their hypothesized causal palaeogeographic processes are temporally congruent [7-9], most vicariance hypotheses are postulated without the benefit of information on the absolute timing of lineage divergences. For instance, on the basis of congruence between phylogenetic and continental break-up patterns, early biogeographic studies attributed disjunct occurrences of Gondwana-distributed taxa to vicariance due to continental drift [10-12]. Similarly, vicariance has traditionally been favored over marine dispersal to explain Gondwanan disjunctions in fish clades whose extant members are obligate freshwater species, such as lungfishes (Dipnoi), bonytongues (Osteoglossiformes), killifishes (Cyprinodontiformes), cichlids (Cichlidae), leaffishes (Nandidae), swamp eels (Synbranchidae), troglobite gobies (Eleotridae), and characins and their allies (Characiformes) [13-19]. Although in these cases vicariance offers the most parsimonious interpretation given the available evidence, temporal discrepancies between the splitting of continentally disjunct lineages and the break-up of Gondwana should not be discarded *a priori*, and if discovered, these would falsify the posited vicariance scenarios. Therefore, time-scaled phylogenies inferred from DNA sequence data using modern analytical methods of molecular dating afford a much-needed means of testing and/or refining such biogeographic hypotheses.

Fishes of the order Characiformes, found throughout much of the freshwaters of the Neotropics and the African continent, are the quintessential transoceanic clade whose present-day distribution has been primarily explained by means of vicariance hypotheses [14,20-23]. A feature common to all of these hypotheses holds that the Early Cretaceous opening of the South Atlantic Ocean is responsible for the disjunct distribution between African citharinoids (suborder Citharinoidei) and the remaining characiform radiation (most of which occurs in the Neotropics). Notwithstanding the growing popularity of molecular dating in phylogenetics, biogeographic hypotheses for the distribution of characiform fishes have barely been tested with temporal information from time-scaled molecular phylogenies. In fact, only the studies of Arroyave and Stiassny [24] and Goodier et al. [25] have implemented molecular clocks to investigate the timing of diversification in a clade of characiform fishes. These studies, nonetheless, were primarily focused on the African family Alestidae and the alestid genus *Hydrocynus*, respectively. Despite some caveats, the chronogram inferred by Arroyave and Stiassny suggests that the origins of characiforms might be too recent for the African/South American drift-vicariance event to adequately explain the split between citharinoids and its Afro-Neotropical sister clade. Under this novel biogeographic scenario, explaining the distribution of extant characiform lineages must recourse to dispersalist arguments.

To further advance our understanding of the chronological framework of characiform evolution, this study investigates the temporal context of citharinoid diversification using DNA sequence data and a Bayesian approach to divergence time estimation in a phylogenetic context. Because citharinoids constitute the sister lineage to the entire remaining Afro-Neotropical characiform radiation [20-22,26-30] (although see [31-33]), a time-calibrated phylogeny of the Citharinoidei has potential to assess the adequacy of current vicariance hypotheses and thus to shed critical light on the early biogeographic history of characiform fishes. Additionally, a comparative examination of the inferred citharinoid evolutionary timescale and the timing of palaeogeological and palaeogeographic events on continental Africa (e.g., the development of contemporary riverine networks) may shed light on the historical processes influencing diversification in citharinoids fishes and other taxa with similar biogeographic patterns. This work will in turn further inform a growing body of biogeographic scenarios proposed to explain current patterns of diversity in African freshwater fishes [24,25,34], most of which rest on the idea that phylogeographic patterns in continental ichthyofaunas are expected to reflect patterns of drainage isolation resulting from landscape evolution [35-37]. Last but not least, given that the only comprehensive phylogenetic treatment of the Citharinoidei is a morphology-based analysis published over three decades ago [26], a molecular phylogeny of citharinoid fishes represents, in and of itself, an imperative endeavor and a significant contribution to the systematics of the poorly studied African ichthyofauna [15,38].

### Diversity and historical overview of citharinoid systematics

**Figure 1:**
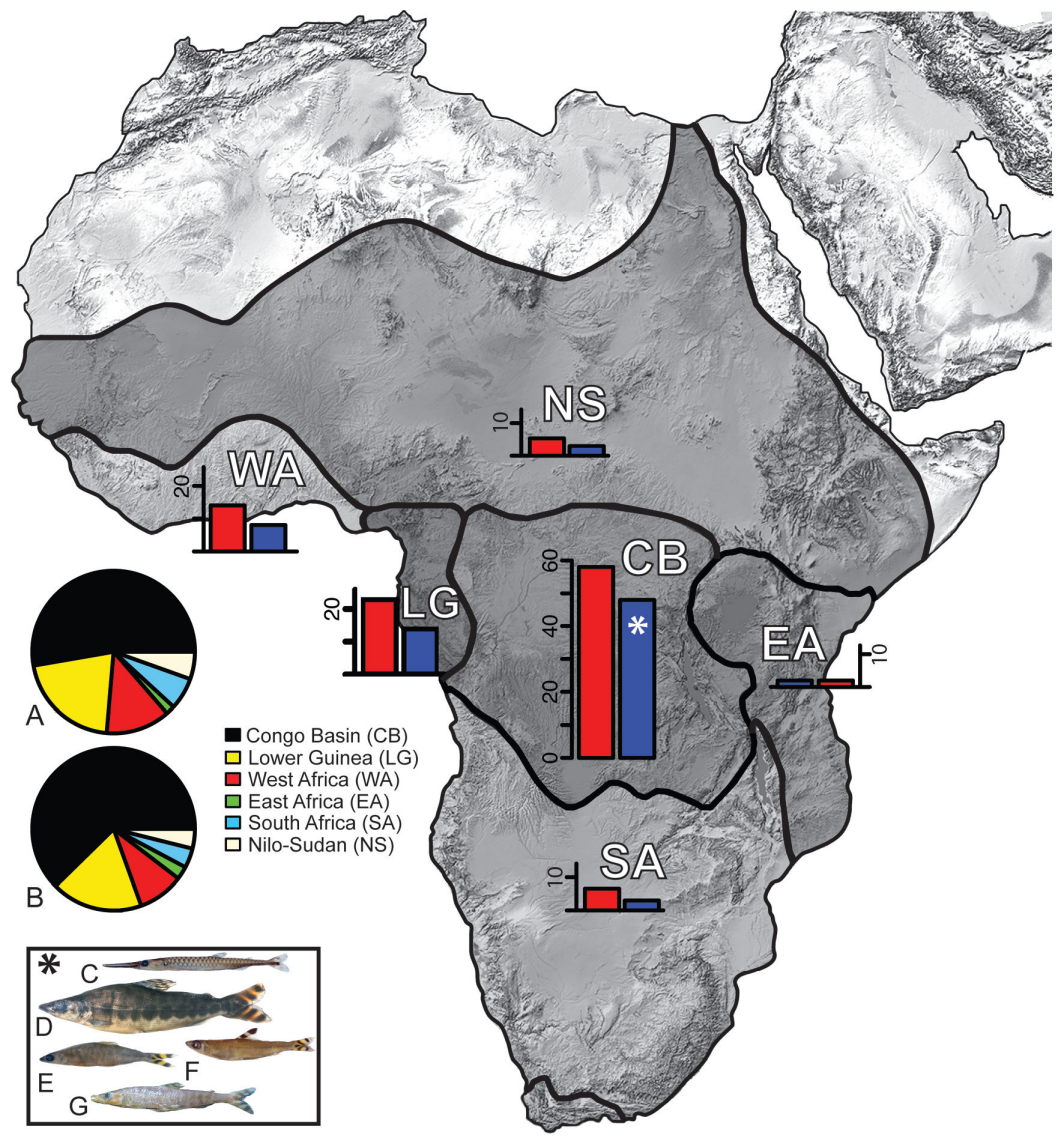
Distichodontid species diversity partitioned by geographic region. CB=Congo Basin, EA=East Africa, NS=Nilo-Sudan, LG=Lower Guinea, SA=South Africa, and WA=West Africa. Inset bar charts indicate number of species present (red) and number of species endemic (blue) to each region. Inset pie charts indicate species occurrences (A) and species endemism (B) across African regions. Inset box shows genera endemic to the Congo Basin: *Belonophago* (C), *Eugnathichthys* (D), *Microstomatichthyoborus* (E), *Hemistichodus* (F), and *Paraphago* (G).

Citharinoid fishes comprise two reciprocally monophyletic families: the Citharinidae, with eight species arrayed in three genera, and the much more speciose Distichodontidae, currently estimated at 96 species arrayed in 15 genera [21,26,39]. Citharinids (commonly known as lutefishes), although not as morphologically and taxonomically diverse as distichodontids, are distributed throughout much of tropical Africa, and constitute an important component of the artisanal fisheries in the region [40]. Similarly, members of the Distichodontidae occur throughout the freshwaters of much of sub-Saharan Africa and the Nile River basin. Despite their pan-African distribution, distichodontids are far from evenly spread across the continent, with species richness heavily concentrated in West-Central Africa and steeply attenuated with distance to the north, east and south. Likewise, levels of species endemism in distichodontids are centered in the Congo Basin, where, in addition to representation of all but one genus (the West-African and Nilo-Sudanic *Paradistichodus*), five genera are endemic (Figure 1). While not as speciose as some other characiform families (such as the African Alestidae or the hyperdiverse Neotropical Characidae), distichodontids exhibit noteworthy morphological variation—particularly in jaw anatomy and dentition—that is reflected in diversified trophic ecologies, ranging from herbivory to carnivory, and including highly specialized ectoparasitic fin-eating behaviors [41,42]. Body size variation is equally noteworthy, with records of total length spanning from less than 2 cm (in certain *Neolebias* species) to over 80 cm (in large *Distichodus* species), and anecdotal reports suggesting that *D. nefasch* can reach over a meter in length. Based on a combination of aspects of jaw morphology and overall body size and shape, distichodontids have been traditionally divided into two evolutionary grades: micropredators and herbivorous species with variously modified jaws and highly variable body plans (as in *Neolebias* and *Distichodus*), and carnivorous or ectoparasitic species with highly kinetic upper jaws, specialized dentition, and elongate bodies (as in *Eugnathichthys* and *Belonophago*) [26,43].

**Figure 2:**
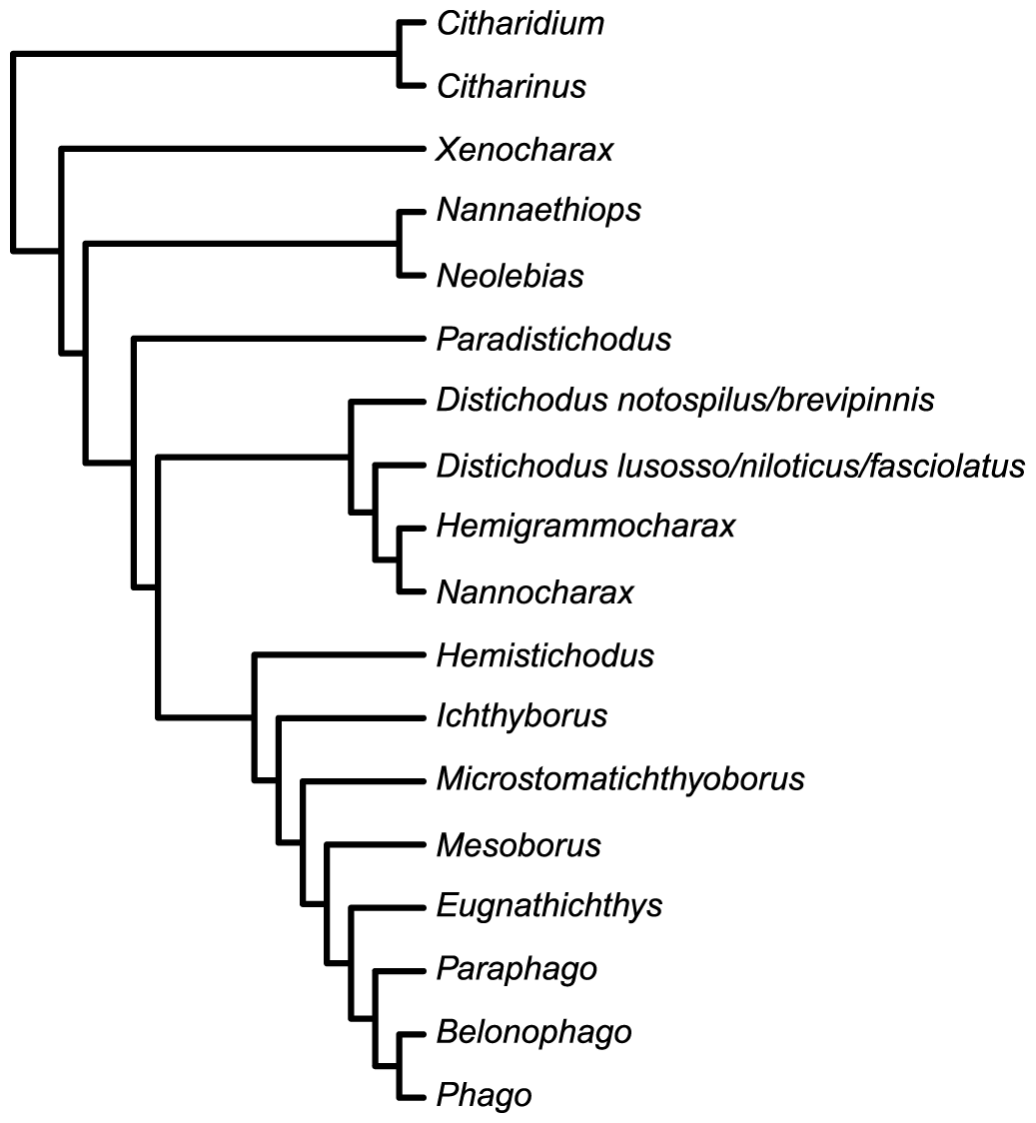
Intergeneric relationships among distichodontid genera as proposed by Vari [25].

The earliest taxonomic treatments of the Citharinoidei date to Boulenger [44] and Eigenmann [45], who divided the assemblage into three and five subfamilies, respectively. Like Eigenmann, Regan [46] recognized five subfamilies, but with different generic composition and limits. Later, Gregory and Conrad [47] expanded the subfamily Citharininae by including the distichodontid genera *Nannaethiops*, *Neolebias*, *Xenocharax* and *Hemistichodus*. In subsequent works, both Monod [48] and Greenwood et al. [49] recognized three subunits within citharinoids. Monod [48], however, did not assign the genera *Neolebias*, *Nannaethiops*, *Xenocharax* and *Paradistichodus* to any of these subunits. In contrast, Greenwood et al. [49] retained membership of Boulenger’s subfamilial groupings while elevating them, for the first time, to familial taxonomic rank. More recently, Poll [50] restricted citharinoids to the families Ichthyboridae and Citharinidae, placing all members of the Distichodontidae (sensu Greenwood et al.) into the latter family. Subsequently, Vari [26] presented a phylogeny (Figure 2) based on osteology and soft anatomy across a comprehensive taxon sampling that included representatives of all distichodontid genera and two of the three citharinid genera (i.e., *Citharinus* and *Citharidium*). It was not until Vari’s study that a classification of citharinoid fishes claimed to reflect evolutionary relationships inferred using cladistic methodology. Prior to this landmark contribution, generic and suprageneric groupings within citharinoids were defined on the basis of plesiomorphic (or combinations of plesiomorphic and derived) characters that failed to define monophyletic groups. Overall, Vari’s findings supported the hypothesis that citharinids and distichodontids are sister taxa, and together constitute the sister clade to the remaining characiform radiation. Nevertheless, previous hypotheses of intergeneric relationships were not entirely supported by his study, requiring a rearrangement of the suprageneric taxonomy of the group. Specifically, the Ichthyboridae (sensu Greenwood et al.) was synonymized with the Distichodontidae, the genera *Congocharax* and *Dundocharax* were synonymized with *Neolebias*, and the genera *Gavialocharax* and *Phagoborus* were synonymized with *Ichthyborus*. Whereas the monophyly of *Distichodus*, *Nannocharax*, or *Hemigrammocharax* was not supported in his study, Vari refrained from making nomenclatural changes regarding those taxa pending analyses based on more comprehensive sampling of species within those genera.

Despite the fact that more than three decades have passed since the publication of Vari’s phylogenetic treatment of the Citharinoidei, there has been no attempt at testing his results, either with molecular or novel morphological data. Therefore, this study is also aimed at providing a comprehensive molecular phylogeny for citharinoid fishes (with emphasis on the Distichodontidae) to further investigate intergeneric relationships and update the evolutionary framework that has laid the foundations for the current classification of the group.

### Molecular dating considerations

Although molecular dating methods have proven fruitful in addressing manifold questions in phylogenetics and evolutionary biology [51,52], these methods require accurate estimates of substitution rates such that genetic distances among taxa can be reliably translated into absolute times of divergence [53]. In cases where an independently estimated substitution rate is unknown (which is mostly the case), the age of one or more internal nodes is needed to calibrate rates of molecular divergence. Such node ages are normally obtained from paleontological evidence, or when such material is unavailable, from dated biogeographic events and/or divergence-time estimates from previous studies. In a recent study aimed at providing a timed-scaled phylogeny of all ray-finned fishes (Actinopterygii), Near et al. [27] suggested that their inferred node ages may be used to calibrate molecular clocks for actinopterygian lineages at lower taxonomic levels (e.g., families) that lack a fossil record. Therefore, another objective of the present study is to assess the suitability of Near et al’s divergence-time estimates as calibration data when dating phylogenies of actinopterygian fishes such as the Citharinoidei.

In principle, one of the virtues of Bayesian inference methods of molecular dating is that these can, to some extent, account for the inherent uncertainty of fossil-based calibrations by incorporating prior knowledge in the form of probability distributions. In practice, however, justification is rarely provided for values assigned to the parameters (technically known as *hyperparameters*) that describe the shape of probability density functions used as priors [54,55]. This is particularly troubling given that hyperparameter choice—and therefore the shape of probabilistic priors—can have a major impact on divergence time estimation [54,56,57]. Although a hierarchical Bayesian model recently proposed by Heath [54] offers a promising avenue toward a less biased choice of hyperparameter values, the author herself acknowledged that modeling hyperparameter uncertainty with hyperpriors such as the Dirichlet distribution does not necessarily represent a biologically explicit approach. Moreover, Heath’s model is currently limited to exponentially distributed priors and its analytical implementation is not readily available in Bayesian molecular dating programs (e.g., BEAST). Given that a standard protocol to properly specify parameters of calibration priors has yet to be proposed, a final objective of this study is to empirically assess the impact of different calibration strategies (particularly the shape of calibration prior densities), as well as the impact of using non-informative priors for the parameters of the clock, speciation, and substitution models, on divergence-time estimates. Thus, the robustness of node ages in the presence of analytical uncertainty can be considered when discussing the biogeographic implications of our findings.

## Materials and Methods

### Ethics Statement

This research was conducted under the American Museum of Natural History (AMNH) Institutional Animal Care and Use Committee (IACUC) approval #36/06. Fishes were collected and euthanized prior to preservation in accordance with established guidelines for the use of fishes in research. Stress and suffering was ameliorated by minimizing handling and through the use of anesthetics prior to euthanasia. Voucher specimens examined in this study were loaned and used with permission from the loaning museums/institutions.

### Taxon sampling

Ingroup taxa included representation of all valid citharinoid genera, with the exception of the monotypic *Citharidium*, *Citharinops*, and *Paraphago*, from which tissue samples were unavailable. Apart from *Paradistichodus* and *Mesoborus*, genera currently considered monotypic [58], and *Microstomatichthyoborus*, for which individuals of only one of two described species were available, all sampled genera were minimally represented by two species. The overall ingroup sampling consisted of 55 valid species (three of the Citharinidae and 52 of the Distichodontidae), thereby encompassing 37.5% and 54.2% of citharinid and distichodontid species diversity, respectively. Where available, multiple individuals per species were included, and sampling of multiple individuals of *Paradistichodus dimidiatus* and *Mesoborus crocodilus* allowed for testing the monophyly of these putatively monotypic genera. In addition to increasing geographic sampling, sequencing of multiple individuals per species allowed for an improved control of sequence quality and recognition of potential contamination issues.

Outgroup taxa comprised representatives of the families Ictaluridae (*Ictalurus punctatus*), and Cyprinidae (*Danio rerio*), both members of otophysan orders closely related to the Characiformes. Outgroup choice was informed by previous studies of characiform and ostariophysian relationships [20-22,26-30], all of which strongly support the monophyly of both the Characiformes and the Citharinoidei, as well as a sister-group relationship between citharinoids and a clade containing the remaining members of the order.

Tissues were obtained primarily from specimens collected during the NSF-funded Congo Project (http://research.amnh.org/vz/ichthyology/congo/index.html) and/or recent fieldwork in West and West-Central Africa. Additional tissues were obtained through donations from colleagues at the Cornell Museum of Vertebrates (USA), Texas A&M University Corpus Christi (USA), and the Royal Ontario Museum (Canada). Fishes were collected and euthanized prior to preservation in accordance with recommended guidelines for the use of fishes in research [59]. Stress and suffering was ameliorated by minimizing handling and through the use of anesthetics prior to euthanasia. This research was conducted under the American Museum of Natural History (AMNH) Institutional Animal Care and Use Committee (IACUC) approval #36/06.

Taxonomically verified vouchers are deposited in the American Museum of Natural History’s ichthyological collection, available online at the museum’s Vertebrate Zoology Collection Database (http://entheros.amnh.org/db/emuwebamnh/index.php) and the Cornell University Museum of Vertebrates (CUMV) Ichthyology Collection (http://testcontent.ornith.cornell.edu/collections/vertebrate/fishes). Species identity of all loaned tissue vouchers was confirmed either by our own examination of loaned voucher specimens (with permission from their respective collection-holding institutions) or on taxonomic authority of the loaning institution. Overall, DNA sequence data was obtained from a total of 121 individuals. Voucher catalog numbers and GenBank accession numbers for the gene sequences generated and included in this study are listed in Table S1.

### Marker selection and character sampling

Nuclear and mitochondrial genes, spanning a range of substitution rates, were sampled for phylogenetic analyses. In total, seven protein-coding genes/gene fragments comprise the comparative data of this study. Nuclear markers consisted of myosin-heavy polypeptide 6-cardiac muscle-alpha (*myh6*), SH3 and PX domain-containing 3-like protein (*sh3px3*), ectodermal-neural cortex 1 (*enc1*), and glycosyltransferase (*glyt*), all of which were originally proposed by Li et al. [60] as promising markers for use in molecular systematics of actinopterygian fishes, and have since been successfully employed in empirical phylogenetic studies of characiform fishes and other ostariophysians [24,61,62]. Mitochondrial markers consisted of cytochrome c oxidase subunit 1 (*co1*), cytochrome b (*cyt-b*), and NADH dehydrogenase 2 (*nd2*), each of which has proven useful in resolving relationships of characiform fishes at multiple phylogenetic levels [21,24,63].

### DNA extraction, amplification and sequencing

General procedures for DNA extraction, amplification, and purification, along with primers and thermal profiles for sequencing *myh6*, *sh3px3*, and *co1*, follow Arroyave and Stiassny [24]. Primer sequences and PCR profiles for *enc1*, *glyt*, cyt-*b*, and *nd2* are listed in Table 1. Distichodontid-specific primers for cyt-*b* and *nd2* were designed on conserved flanking regions for each fragment using Primer3 [64]. Contig assemblage and sequence editing was performed using Geneious Pro version 5.6.5 (Biomatters, available from http://www.geneious.com/). IUPAC nucleotide ambiguity codes were used to represent heterozygous sites.

**Table 1:** Primers and PCR profiles for amplification of *enc1*, *glyt*, cyt-*b*, and *nd2*.

**Gene**	**Source**	**Primer**	**Primer Sequence^a^**	**PCR Thermal Profile^b^**
*enc1*	Li et al. [53]	ENC1_F85	5'-GACATGCTGGAGTTTCAGGA-3'	(98 °C/20s, 57 °C/30s, 72 °C/45s) x 25 + (98 °C/20s, 55 °C/30s, 72 °C/45s) x 10
		ENC1_R982	5'-ACTTGTTRGCMACTGGGTCAAA-3'	
		ENC1_F88^c^	5'-ATGCTGGAGTTTCAGGACAT-3'	(98 °C/20s, 57 °C/30s, 72 °C/45s) x 25 + (98 °C/20s, 55 °C/30s, 72 °C/45s) x 10
		ENC1_R975^c^	5'-AGCMACTGGGTCAAACTGCTC-3'	
*glyt*	Li et al. [53]	Glyt_F559	5'-GGACTGTCMAAGATGACCACMT-3'	(98 °C/20s, 57 °C/30s, 72 °C/45s) x 25 + (98 °C/20s, 55 °C/30s, 72 °C/45s) x 10
		Glyt_R1562	5'-CCCAAGAGGTTCTTGTTRAAGAT-3'	
		Glyt_F577^c^	5'-ACATGGTACCAGTATGGCTTTGT-3'	(98 °C/20s, 57 °C/30s, 72 °C/45s) x 25 + (98 °C/20s, 55 °C/30s, 72 °C/45s) x 10
		Glyt_R1464^c^	5'-GTAAGGCATATASGTGTTCTCTCC-3'	
*cyt-b*	This study	*cyb*_Dist_f	ACAGGTCTTGGTTAGARTCCRGGYGGG	(95 °C/60s, 58 °C/60s, 72 °C/120s) x 35
		*cyb*_Dist_r	CCGGATTACAAGACCGGCGCT	
*nd2*	This study	*nd2*_Dist_f	AGCTTTTGGGCCCATACCCCA	(95 °C/60s, 58 °C/60s, 72 °C/120s) x 35
		*nd2*_Dist_r	AGGRACTAGGAGATTTTCACTCCTGCT	

^a^ Listed from 5’ to 3’.

^b^ Conditions for denaturation, annealing and extension steps for each cycle are listed in parenthesis, followed by the number of cycles. All reactions included a 5-minute initial denaturation at 95°C and a 7-minute final extension at 72°C.

^c^ Primers used during a second (nested) PCR, required for successful amplification; 1:20 dilution between rounds.

### Alignment and model selection

Each gene partition was aligned based on the translated amino acid sequence using the *Translation Align* algorithm under default parameters, as implemented in Geneious Pro version 5.6.5. The number of variable and parsimony-informative sites of the concatenated alignment was determined using MEGA 5 [65]. Nucleotide substitution model selection for each gene partition was accomplished by means of the Bayesian Information Criterion (BIC) as implemented in jModelTest [66] under the following likelihood settings: *Number of substitution schemes* = 3; *Base frequencies* = +F; *Rate variation* = +I and +G with nCat = 4; and *Base tree for likelihood calculations* = Fixed BIONJ-JC, so that a total of 24 models were evaluated.

### Assessment of substitution saturation

Third codon positions of each gene partition were checked for substitution saturation using both Xia et al.’s test [67] and saturation plots (i.e., observed number of transitions and transversions against corrected genetic distance for all pairwise comparisons among terminals), following the guidelines provided in [68]. In saturation plots, corrected genetic distances were calculated based on the best-fit substitution models previously determined by jModelTest, and trend lines were estimated using second-order polynomial curves fit to the data. Both approaches were implemented in DAMBE [69]. Because of the limited number of available substitution models in DAMBE, corrected genetic distances were calculated using F84 as an alternative for HKY+I+G and GTR as an alternative for SYM+I+G and GTR+I+G. Saturation in a data partition was assumed when the index of substitution saturation (*I*_*SS*_) was either larger or not significantly smaller than the critical value (*I*_*SS.C*_) and/or transversions outnumbered transitions in saturation plots.

### Phylogenetic reconstruction

The concatenated alignment was analyzed using both statistical (model-based) and parsimony methods of phylogenetic inference. Statistical approaches to phylogeny estimation included both frequentist (likelihood) [70] and Bayesian (posterior probability) [71] inference methods. To accommodate potential process heterogeneity among gene regions, model-based analyses were conducted on the concatenated alignment partitioned into gene regions with parameters unlinked. Likelihood analyses were carried out in RAxML version 7.2.8 Black Box [72]. Bayesian inference of phylogeny was carried out in MrBayes version 3.1.2 [73,74] and implemented using the Markov Chain Monte Carlo algorithm (MCMC) run for 5 × 10^7^ generations with a sampling period of 1000 generations, under default priors and default proposal mechanisms. A total of two independent runs of four chains each were performed. Convergence of the MCMC algorithm to a stationary distribution—and thus the number of generations to be discarded as burn-in—was determined by examination of trace plots of posterior probability vs. number of generations using Tracer [75]. Graphical exploration of MCMC runs was also achieved by plotting posterior probabilities of splits at selected increments over an MCMC run (cumulative function) using the web-based tool AWTY [76,77]. Further assessment of MCMC convergence was undertaken by examination of the average standard deviation of split frequencies, with values << 0.01 taken as indicative of stationarity. Accordingly, 25% of MCMC samples were discarded as burn-in, and substitution model parameters were calculated from the remaining 75%. Likewise, branch lengths and posterior probabilities of nodes were calculated from the set of post burn-in trees using TreeAnnotator version 1.7.4 [78] and summarized as a 50% majority rule consensus tree. Both RAxML and MrBayes analyses were implemented through the CIPRES Science Gateway V. 3.3 [79].

Parsimony analyses were carried out using TNT (Willi Hennig Society edition) [80,81] with gaps treated as fifth state, an indel-substitution cost ratio of 1, and no cost for gap opening. The heuristic tree search strategy in TNT included 1000 Wagner trees [82], tree bisection and reconnection (TBR) branch swapping [83], perturbation using the Parsimony Ratchet [84], and tree fusing [85]. Branches with zero possible length were collapsed. Ensemble consistency index (CI) [86] and ensemble retention index (RI) [87] were used as measures of homoplasy and synapomorphy, respectively.

In likelihood and parsimony analyses, nodal support was estimated by means of the bootstrap character resampling method [88,89] using 1000 pseudoreplicates, whereas in Bayesian analyses nodal support was assessed using clade posteriors. When detected, saturated positions were removed from the data so as to produce an alternative, more restricted dataset, which was analyzed in the same manner as the original dataset. All resultant phylogenies were rooted at *Danio rerio*.

### Bayesian estimation of divergence times

Node ages were estimated using a Bayesian relaxed-clock method [90] under the uncorrelated lognormal (UCLN) rate variation model as implemented in BEAST version 1.7.4 [78,91]. A Yule process prior for topology and divergence times was assumed. Justification for the use of both the UCLN relaxed-clock model and the Yule process prior is provided in Arroyave and Stiassny [24]. Both primary (i.e., fossil-based) and secondary (i.e., based on age estimates from previous molecular dating studies) calibrations were used to estimate substitution rates and eventually absolute times of divergence. Primary calibrations were incorporated using log-normally distributed priors based on the oldest fossils assignable to members of the suborder Citharinoidei; namely, Eocene (*ca* 46 Ma) fossil remains corresponding to † *Eocitharinus macrognathus* [92] and Late Miocene (*ca* 7.5 Ma) fossilized dentition attributable to *Distichodus* [93]. † *Eocitharinus macrognathus* was described from a compression fossil of a partial skeleton (part and counterpart of the anterior two-thirds of the body) from the Eocene Mahenge site in north-central Tanzania, whose absolute age has been dated to 45-46 Ma [94]. While † *Eocitharinus macrognathus* exhibits several features that suggest a close association with the Citharinidae and Distichodontidae (e.g., prominent lateral ridge on the anterodorsal corner of the opercle, fused postcleithra 2+3), it lacks determinable synapomorphies of either family and has been thereby classified as Citharinoidei *incertae sedis* [23,92]. Therefore, † *Eocitharinus macrognathus* was incorporated for calibration purposes as a stem member of the Citharinoidei. The other calibration fossil used corresponds to the oldest from a series of Neogene *Distichodus* remains. This *Distichodus* fossil consists of a single tooth recovered from Late Miocene fluvial strata of the Lower Nawata (7.44 ± 0.05 Ma) formation, Lothagam, Kenya [93,95-97]. *Distichodus* dentition recovered at Lothagam is diagnostic of the genus and corresponds to a fish estimated to be of up to a meter long and therefore similar in size to certain extant species from the region [93]. Absolute age estimates of † *Eocitharinus macrognathus* and *Distichodus* fossils were used to calibrate the nodes (as minimum age constraints) corresponding to the most recent common ancestor (MRCA) of citharinoids (as a stem lineage) and the MRCA of the genus *Distichodus*, respectively (Table 2). Secondary calibrations were incorporated using normally distributed priors based on divergence-time estimates imported from a recent study on the timing of diversification of ray-finned fishes by Near et al. [27]; specifically, the estimated ages of the MRCA of the Otophysi (170 Ma; 95% HPD=185-155) and the MRCA of Characiformes and Siluriformes (130 Ma; 95% HPD=140-120). The standard deviation (σ) parameter of the normal distribution associated with these secondary calibrations was chosen so that 95% of the probability lay within the boundaries of the 95% HPD intervals recovered by Near et al. (see Table 2).

**Table 2:** Prior distributions and parameter settings of calibration nodes.

**Node**	**Log-normal 95th percentile soft max. bound**	**BEAUti Settings**
MRCA Otophysi [46]	n/a	Normal (*Initial Value*: 170; μ=170; σ=9.1)
MRCA Siluriformes+Characiformes [46]	n/a	Normal (μ=130; σ=6.1)
MRCA Citharinoidea [85]	"Young" = 52.83 Ma	LogNormal (μ=1.1; σ=0.5; offset: 46)
	"Intermediate" = 80.14 Ma	LogNormal (μ=2.7; σ=0.5; offset: 46)
	"Old" = 107.45 Ma	LogNormal (μ=3.3; σ=0.5; offset: 46)
MRCA *Distichodus* [86]	"Young" = 9.5 Ma	LogNormal (μ=0; σ=0.5; offset: 7.5)
	"Intermediate" = 17.54 Ma	LogNormal (μ=1.6; σ=0.5; offset: 7.5)
	"Old" = 25.58 Ma	LogNormal (μ=2.2; σ=0.5; offset: 7.5)

**Figure 3:**
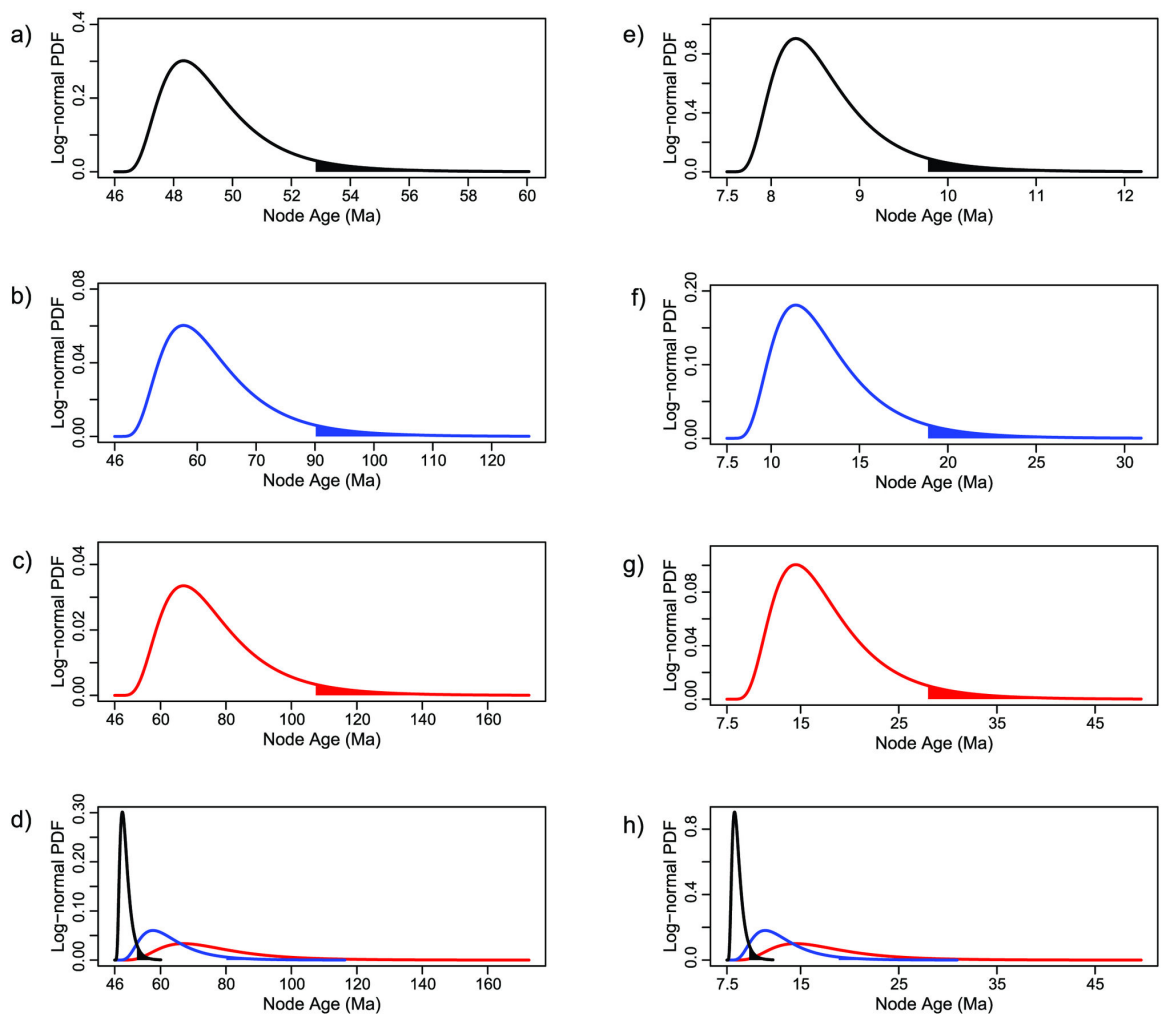
Alternative priors of fossil-calibrated nodes used in the sensitivity analysis. Log-normally distributed calibration priors for the age of the MRCA of Citharinoidei (a-d) and *Distichodus* (e-h) as plotted separately and differentially scaled (a-c, e-g), and combined and equally scaled (d, h). The lower limit of the x-axis interval defining the area shaded under the curves corresponds to the 95^th^ percentile soft maximum bound of each calibration prior.

In log-normally distributed calibration priors, the larger the mean (μ), the flatter the probability density function, and thereby the older the 95^th^ percentile soft maximum bound for the age of the node. Accordingly, the robustness of inferred node ages to changes in the shape of log-normally distributed priors was assessed by conducting a series of analyses (in the manner of sensitivity analysis) where the standard deviation hyperparameter was fixed (σ=0.5) but μ was allowed to vary so that three alternative priors (arbitrarily assigned as “young”, “intermediate”, and “old”) were considered at each calibration node (Figure 3; Table 2). These alternative priors, and thus their corresponding μ, were formulated to be consistent with molecular clock- and fossil-based age estimates of clades bracketing each calibration node. Specifically, the proposed alternative priors for the age of the MRCA of the Citharinoidei node were devised in accordance with the estimated age of the MRCA of the Characiformes (*ca* 90-100 Ma) [24,27] and the stratigraphic distribution of fossils unambiguously assignable to the order [23]. Similarly, the proposed alternative priors for the age of the MRCA of *Distichodus* were formulated to be compatible with paleontological evidence [93,97] and the chronogram proposed by Arroyave and Stiassny [24]. Moreover, because the distinctive character(s) that assign a fossil to a given taxon might have evolved along the stem lineage [98], an additional prior for the age of the MRCA of *Distichodus* in which the fossil was treated as a stem member (as opposed to crown member; the default option in BEAST) was considered.

The effect of using non-informative priors for the parameters of the UCLN relaxed clock model (μ and σ), the Yule speciation process (birth rate), and the model of molecular evolution for each gene (substitution rates, base frequencies, gamma shape, and proportion of invariant sites), was similarly assessed by performing analyses using default vs. uniform (thus non-informative) priors. Additionally, the suitability of Near et al.’s inferred node ages [27] as calibration information was explored by comparing the results of an analysis that included both primary and secondary calibrations with those from analyses using either only primary or only secondary calibrations. A detailed description of the analyses conducted to assess the impact of different calibration strategies on divergence-time estimates is presented in Table 3.

**Table 3:** Sensitivity analysis devised to explore the robustness of divergence-time estimates to changes in prior hyperparameters and analysis settings.

**Analysis**	**MRCA Citharinoidea Prior**	**MRCA *Distichodus* Prior**	**Secondary Calibrations**	**UCLN, Yule, and Substitution Model Priors**
1	Intermediate	Intermediate	Yes	Default
2	Intermediate	Young	Yes	Default
3	Intermediate	Old	Yes	Default
4	Young	Intermediate	Yes	Default
5	Old	Intermediate	Yes	Default
6	Intermediate	Intermediate^1^	Yes	Default
7	Intermediate	Intermediate	No^2^	Default
8	No	No	Yes	Default
9	Intermediate	Intermediate	Yes	Uniform
10	Intermediate	Young	Yes	Uniform
11	Intermediate	Old	Yes	Uniform
12	Young	Intermediate	Yes	Uniform
13	Old	Intermediate	Yes	Uniform
14	Intermediate	Intermediate^1^	Yes	Uniform
15	Intermediate	Intermediate	No^2^	Uniform
16	No	No	Yes	Uniform

^1^ Calibrated as stem lineage

^2^ Calibrated using implied tree prior

Each analysis listed in Table 3 consisted of two independent, identical runs, with a chain length of 5 × 10^7^ generations (except for Analyses 2 and 10, which required 2 × 10^8^ to reach convergence), a sampling period of 1000 generations, and default proposal mechanisms. After ensuring that stationarity had been reached, tree files (.trees) of independent (yet identical) runs were combined into a single file using LogCombiner [78], discarding 25% of samples as burn-in. The posterior sample of trees (post burn-in) contained in each combined tree file was then summarized using TreeAnnotator to produce a chronogram indicating posterior probabilities and mean ages of all nodes with their associated 95% highest posterior density (HPD) intervals.

## Results

### Sequence data summary statistics and substitution model selection

The concatenated alignment of all seven genes consisted of 5820 sites, of which 2599 were variable (including 18 indels) and 2234 parsimony-informative. A few instances of failed DNA amplification and/or sequencing resulted in ~4% of missing data. Summary statistics for each individual gene partition and the results of the statistical selection of best-fit models performed in jModelTest are presented in Table 4.

**Table 4:** Summary statistics for each individual gene partition and the results from the statistical selection of best-fit models.

**Gene**	**OTUs coverage (%)**	**AlignmentLength**	**Variable Sites (#)**	**Variable Sites (%)**	**Parsimony-informative Sites (#)**	**Parsimony-informative Sites (%)**	**jModelTest best-fit Model (BIC)**
*co1*	96	657	268	40.8	260	39.5	HKY+I+G
*cyt-b*	92.5	999	495	49.5	459	46	HKY+I+G
*enc1*	93.4	825	283	34.3	231	28	SYM+I+G
*glyt*	97.5	843	377	44.7	299	35.5	K80+I+G
*myh6*	100	795	278	35	208	26.2	GTR+I+G
*nd2*	94.2	981	636	64.8	585	59.6	GTR+I+G
*sh3px3*	98.3	720	262	36.4	192	26.7	K80+I+G

### Substitution saturation

Results of Xia et al’s statistical test of saturation [67] were ambivalent, ultimately dependent on the shape of tree topology. Assuming a perfectly symmetrical topology, third codon positions of all genes resulted in a calculated index of substitution saturation (*I*_*SS*_) significantly lower than the critical value (*I*_*SS.C*_), thus implying little saturation in the data. Under the assumption of a markedly asymmetrical topology, however, third positions of *co1*, cyt-*b*, and *nd2*, resulted in *I*_*SS*_ values significantly higher than their corresponding *I*_*SS.C*_ values, thereby suggesting that these sites have experienced substantial saturation. Conversely, plots of observed number of transitions and transversions against corrected genetic distance (d) (Figure S1) indicated that only third codon positions of the mitochondrial genes cyt-*b* and *nd2* had reached substitution saturation, specifically at genetic distances of approximately 0.26 and 0.29 substitutions/site, respectively (Figure S1). This implies that about 14% of pairwise comparisons might be affected by saturation in third positions of *nd2*, whereas less than 3% of pairwise comparisons might be affected by saturation in third positions of cyt-*b*. Although *co1* comes near to experiencing substitution saturation in third codon positions at a corrected genetic distance of about 0.27 substitutions/site, this has no practical implications since genetic distances of virtually all pairwise comparisons in the dataset fall below this value (Figure S1). Based on the results from both Xia et al’s test and saturation plots, it seems reasonable to assume that only third positions of *nd2* might negatively affect phylogeny estimation due to substitution saturation, although most likely not substantially. Therefore, in addition to the original matrix, an alternative dataset excluding these putatively saturated positions was analyzed, so that potential differences in topology after removal of saturated data could be considered when discussing citharinoid relationships.

### Citharinoid phylogeny

**Figure 4:**
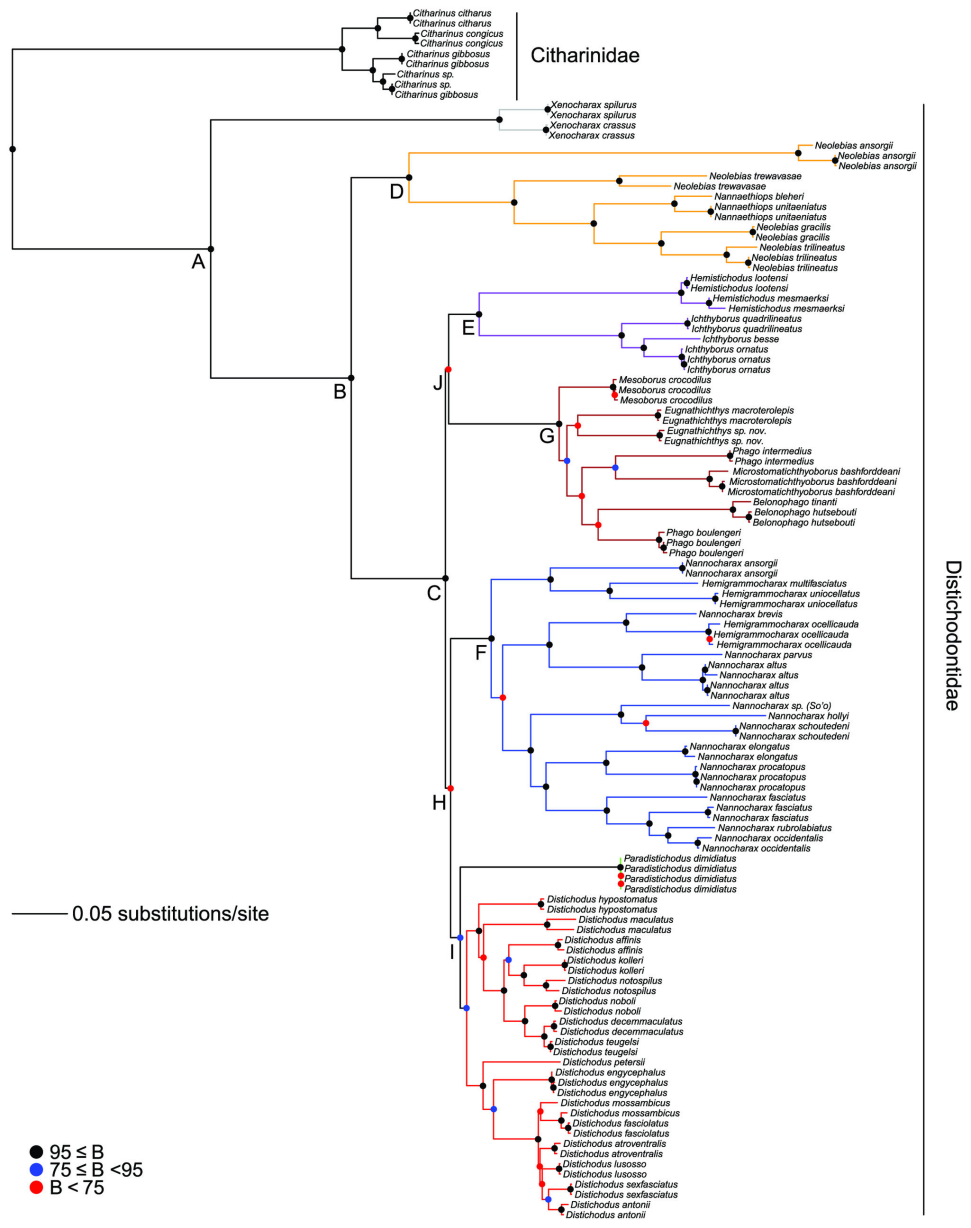
Phylogeny of the Citharinoidei as inferred by likelihood in RAxML. Letters A-J indicate major suprageneric clades. Colored circles on nodes indicate degree of support as determined by bootstrap values (B). Branches of select generic and suprageneric assemblages are differentially colored to indicate composition and configuration of distichodontid subclades discussed in the text. Long branches leading to the outgroup species *Danio rerio* (root) and *Ictalurus punctatus* are not shown.

**Figure 5:**
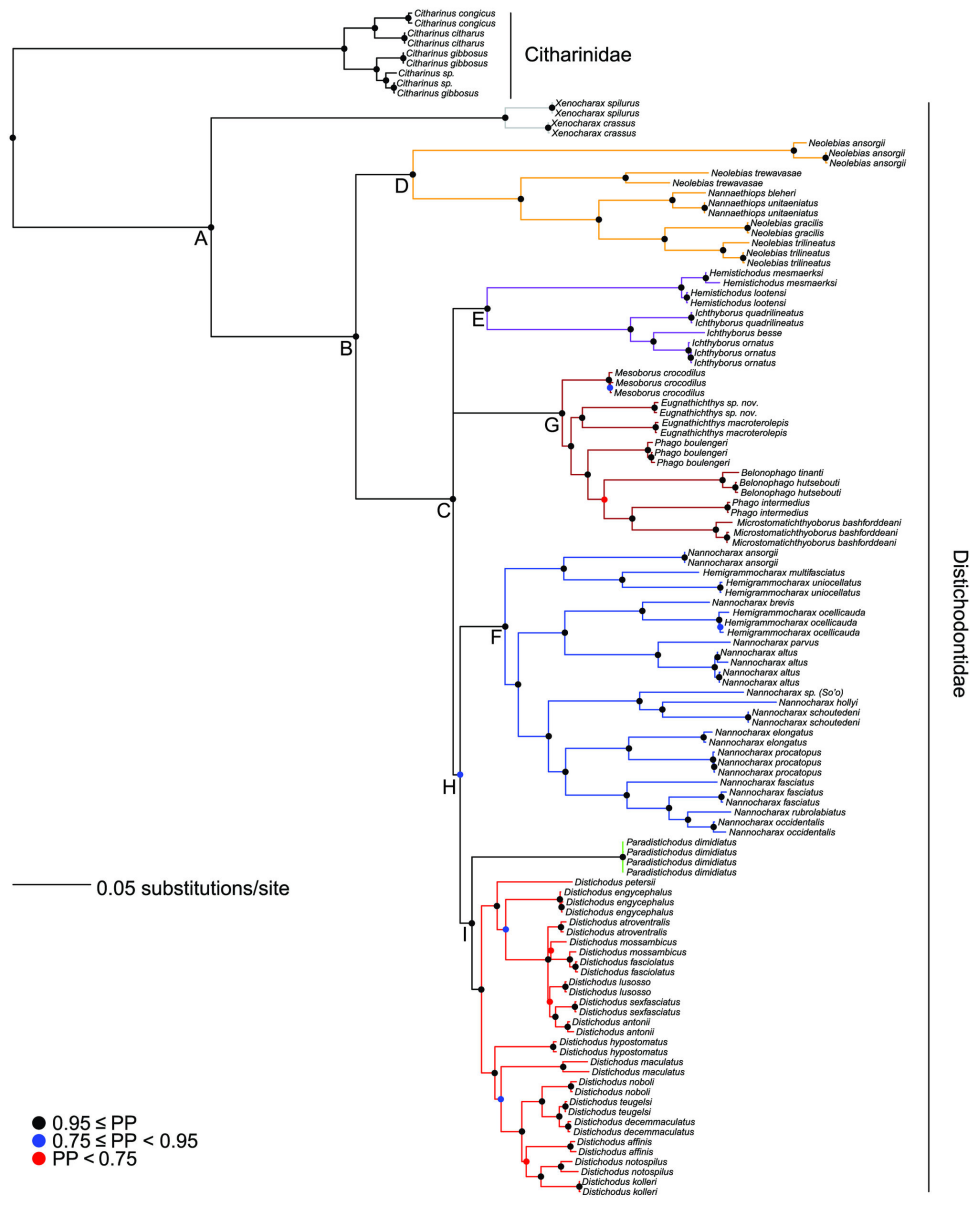
Phylogeny of the Citharinoidei as recovered by Bayesian inference in MrBayes. Colored circles on nodes indicate degree of support as determined by posterior probabilities (PP). Branches and terminals colored as in the RAxML tree (Figure 4).

**Figure 6:**
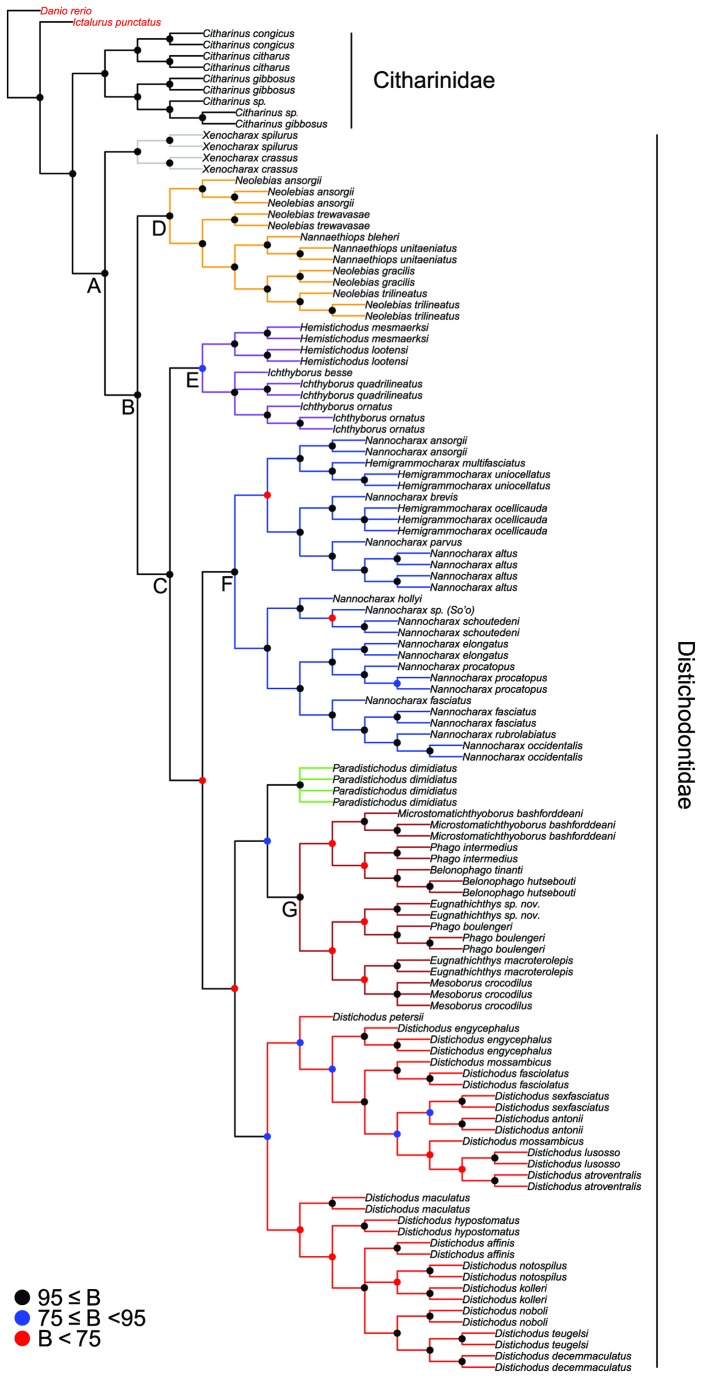
Phylogeny of the Citharinoidei as recovered by parsimony in TNT. The topology corresponds to the strict consensus of 12 equally most parsimonious trees (L=14047; CI=0.272; RI=0.765). Letters A-G indicate major suprageneric clades also recovered by model-based methods. Colored circles on nodes indicate degree of support as determined by bootstrap values (B). Branches and terminals colored as in the RAxML tree (Figure 4). Outgroup taxa in red.

Model-based analyses resulted in likelihood (i.e., RAxML most optimal tree) and Bayesian (i.e., MrBayes 50% majority rule consensus tree) phylogenies of almost identical topology, with similar clade support and relative branch lengths (Figures 4 and 5). These phylogenies are mostly well supported and corroborate the monophyly of the Distichodontidae (Figures 4 and 5). Parsimony analysis resulted in 12 equally most parsimonious trees of length 14047, of which a strict consensus is presented in Figure 6. Most of the very few polytomies in the parsimony strict consensus involve within-species resolution (as expected), whereas only two instances involve failure to completely resolve within-genus relationships, namely in *Ichthyborus* and *Distichodus*. Monophyly of distichodontids was likewise supported by the parsimony topology.

Both model-based and parsimony phylogenies recovered the genus *Xenocharax* as sister to all other distichodontids (node B; Figures 4-6), and a clade consisting of the diminutive genera *Nannaethiops* and *Neolebias* (although not reciprocally monophyletic—node D; Figures 4-6) as sister to the remaining distichodontid radiation (node C; Figures 4-6). Likewise, both approaches revealed a clade containing the reciprocally monophyletic *Ichthyborus* and *Hemistichodus* (node E; Figures 4-6), and a strongly supported suprageneric clade (although not identically resolved) containing ectoparasitic fin-eating (*Belonophago*, *Eugnathichthys*, and *Phago*), ichthyophagous (*Mesoborus*), and micropredatory (*Microstomatichthyoborus*) genera (node G; Figures 4-6). Most suprageneric clades in the model-based phylogenies (nodes B-J in Figure 4 and B-I in Figure 5) are well supported, with only two nodes (H, J; Figure 4) exhibiting bootstrap values below 60 in the likelihood tree.

The parsimony tree differed with the model-based trees mainly in the placement of three clades, namely, the one containing the African darters of the genera *Hemigrammocharax* and *Nannocharax*, the one containing members of *Ichthyborus and Hemistichodus*, and the one containing individuals of the species *Paradistichodus dimidiatus*. Not surprisingly, the nodes involved are in most cases either weakly supported or collapsed (Figures 4-6).

**Figure 7:**
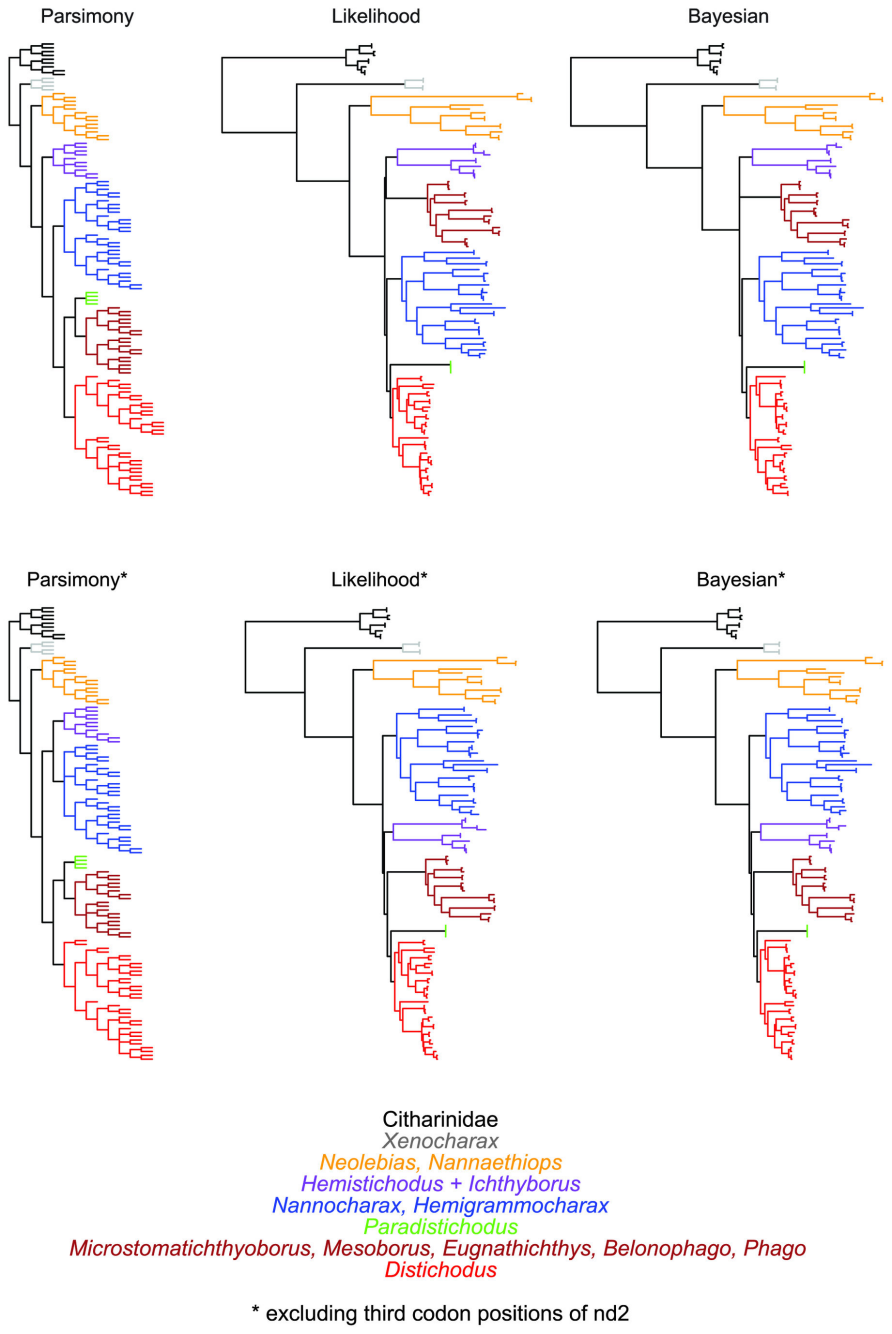
Comparative summary of citharinoid phylogenies inferred with and without putatively saturated sequence data. Parsimony and model-based phylogenies based on the original dataset (top) and a restricted dataset with *nd2* sequence data removed (bottom). Branches are differentially colored by generic/suprageneric subclade, of which the composition is indicated by the genus/genera matching the color of its branches.

Overall, excluding *nd2* third positions did not result in major topological differences, and most of the abovementioned well-supported suprageneric clades (nodes B-J) were similarly recovered. Only the phylogenetic placement of the clades *Nannocharax*+*Hemigrammocharax* and *Ichthyborus*+*Hemistichodus* was influenced by removal of these putatively saturated data. A comparative summary of the results of parsimony and model-based analyses, including those with *nd2* third positions removed, is presented in Figure 7.

### Monophyly of distichodontid genera

Except for the genus *Microstomatichthyoborus* for which tissues from only a single species were available, taxon sampling allowed for testing of generic monophyly in the Distichodontidae. In all trees, regardless of optimality criterion, monophyly of *Belonophago, Distichodus*, *Hemistichodus*, *Ichthyborus*, *Mesoborus, Nannaethiops*, and *Xenocharax* was corroborated (Figures 4-6). Monophyly of *Eugnathichthys*, however, was confirmed in the likelihood and Bayesian trees (Figures 4 and 5) but not in the parsimony tree (Figure 6). By contrast, regardless of inference method applied, no support for the monophyly of *Hemigrammocharax*, *Nannocharax*, *Neolebias*, or *Phago* was found. Placement of *Nannaethiops* well nested within *Neolebias* rendered the latter paraphyletic. Similarly, sampled species of *Hemigrammocharax* were recovered nested within *Nannocharax*, rendering the latter paraphyletic. Perhaps more surprisingly, *Phago* was recovered as polyphyletic by all methods (although differently resolved).

### Timescale of citharinoid diversification

**Figure 8:**
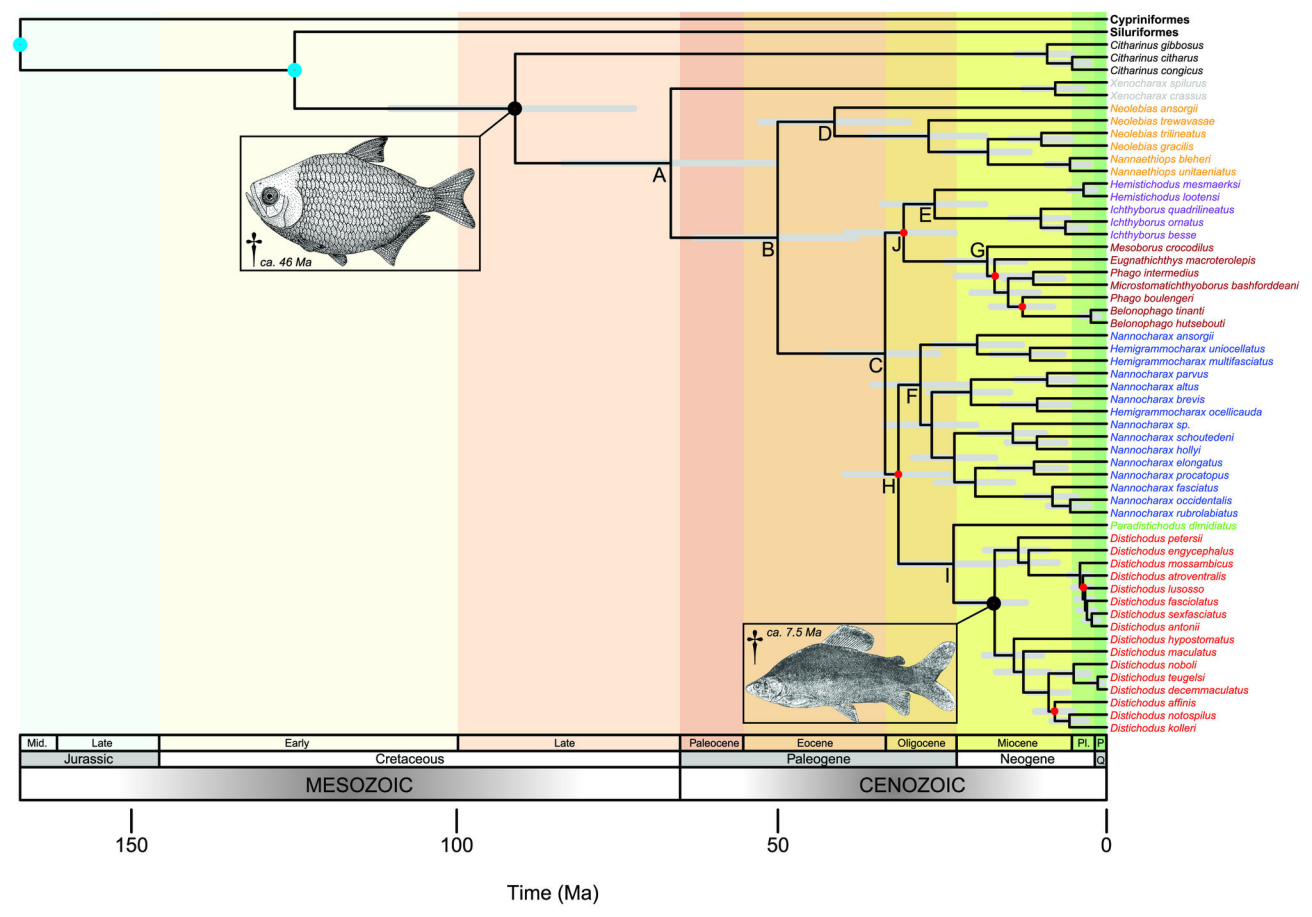
Time-scaled citharinoid phylogeny. This chronogram was inferred using both primary (with “intermediate” priors) and secondary calibrations (Analysis 1; Table 3). Primary calibration nodes are indicated by black dots and linked to a figure representing the fossil. Secondary calibration nodes are indicated by blue dots. Divergence-time estimates are represented by the mean ages of clades. Gray bars correspond to 95% highest posterior density (HPD) intervals of mean node ages. Terminal taxa are colored by generic/suprageneric clade membership, following the color scheme of Figures 4-7. All nodes resulted in posterior probabilities (PP) larger than 0.95, except for those labeled in red, for which PP < 0.75.

A time-scaled phylogeny of the Citharinoidei, inferred using both primary (based on “intermediate” priors) and secondary calibrations (Analysis 1; Table 3), resulted in a topology identical to that of the likelihood tree, and with comparable nodal support (i.e., most clades with posterior probabilities > 0.95) and relative branch lengths (Figure 8). The chronogram indicates that, based on estimated mean node ages, the origins of the Citharinoidei and the Distichodontidae date to the Turonian (90.86 Ma; 95% HPD=110-73) and the Maastrichtian (66.9 Ma; 95% HPD=83-51) of the Late Cretaceous, respectively. However, most modern distichodontid genera, as well as the citharinid genus *Citharinus*, appear to have originated and diversified much more recently, mainly during the Neogene (23-2.6 Ma). For instance, the youngest of the well-supported suprageneric clades recovered—represented by the MRCA of fin-eating distichodontids and allied genera (node G; Figures 4-6)—dates to the Early Miocene (*ca.* 18 Ma).

Further, our results indicate that estimated ages of fossil-calibrated nodes are considerably older than the ages of the calibration fossils. Specifically, the inferred age for the node representing the MRCA of *Distichodus* (17.22 Ma; 95% HPD=23-12) is more than twice as old as the age of the fossil used to calibrate the node (*ca.* 7.5 Ma). Likewise, the inferred age of the node representing the MRCA of the Citharinoidei (90.86 Ma; 95% HPD=110-73) is almost twice as old as the age of the stem citharinoid fossil († *Eocitharinus macrognathus*) used for calibration (*ca.* 46 Ma). By contrast, the inferred ages of the (external) nodes calibrated using Near et al’s divergence-time estimates are very similar to the mean ages proposed in their actinopterygian chronogram. This is, 166.86 Ma [95% HPD=185-150] (vs. 170 Ma) for the node represented by the MRCA of the Otophysi, and 124.73 Ma [95% HPD=137-113] (vs. 130 Ma) for the node represented by the MRCA of characiforms and siluriforms [27].

Results of analyses aimed at assessing robustness of the inferred node ages to changes in calibration settings and parameter priors (Analyses 1-16) are presented in Table 5. By and large, changes to the shape of log-normally distributed priors did not result in substantially different divergence-time estimates. In fact, differential calibration priors for the node representing the MRCA of Citharinoidea (Analyses 1, 4, and 5) resulted in nearly identical estimates. While divergence times based on “intermediate” and “old” calibration priors for the node representing the MRCA of *Distichodus* were likewise very similar (Analyses 1 and 3), using a “young” calibration prior resulted in comparatively younger estimates (Analysis 2). On the other hand, exclusion of secondary calibrations (Analysis 7) had a sizeable impact on inferred node ages, and resulted in estimates almost twice as young as in the control (i.e., Analysis 1). Conversely, exclusion of primary (i.e., fossil) calibrations resulted in the oldest estimates (Analysis 8). Treating the fossil used to calibrate the node representing the MRCA of *Distichodus* as a stem (as opposed to crown) member (Analysis 6) did not result in significantly different divergence-time estimates. Likewise, using uniform instead of default priors for the parameters of the UCLN relaxed clock model, Yule process, and substitution models (Analyses 9-16) resulted in negligible differences in the estimated distichodontid node ages.

**Table 5:** Estimated mean ages^*^ and associated 95% HPD intervals of select nodes from the sensitivity analysis chronograms.

**Analysis**	**Otophysi^1^**	**MRCA of Characiformes and Siluriformes^1^**	**Citharinoidea^2^**	**Distichodontidae**	***Distichodus*^2^**
1	166.86 (185-150)	124.73 (137-114)	90.86 (110-73)	66.90 (84-51)	17.22 (23-12)
2	158.59 (180-130)	122.27 (134-111)	76.92 (103-47)	54.86 (74-34)	11.68 (16-18)
3	166.33 (185-148)	125.93 (138-114)	94.23 (113-76)	69.96 (86-54)	18.85 (24-14)
4	166.17 (183-149)	121.89 (133-109)	90.18 (109-72)	66.19 (83-51)	16.96 (22-12)
5	166.33 (185-149)	126.07 (138-115)	92.11 (111-73)	67.88 (84-51)	17.39 (23-12)
6	165.78 (184-148)	124.92 (137-113)	91.46 (111-72)	67.35 (84-51)	17.21 (23-13)
7	68.48 (94-51)	60.32 (73-50)	46.78 (59-36)	35.38 (45-26)	10.82 (13-19)
8	167.1 (185-149)	130.58 (142-119)	99.08 (118-79)	74.63 (92-57)	21.87 (29-15)
9	166.37 (183-148)	125.01 (137-113)	91.31 (110-73)	67.55 (84-52)	17.4 (23-12)
10	158.25 (180-130)	122.31(135-111)	76.97 (103-47)	55.01 (74-34)	11.67 (16-18)
11	166.18 (186-148)	125.87 (137-114)	94.67 (114-76)	70.26 (88-54)	18.9 (25-13)
12	166.18 (183-149)	121.97 (134-110)	89.82 (108-71)	66.48 (82-50)	16.92 (22-12)
13	166.18 (184-149)	126.15 (138-115)	93 (112-74)	68.48 (85-53)	17.52 (23-12)
14	166.2 (185-149)	124.78 (137-113)	91.25 (110-72)	67.27 (83-50)	17.62 (23-13)
15	68.71 (93-50)	60.3 (73-50)	46.55 (59-36)	34.88 (45-26)	10.79 (13-18)
16	167.31 (185-149)	130.37 (142-119)	99.24 (118-79)	74.99 (92-58)	21.85 (29-15)

* Node ages in Ma.

^1^ Primary-calibrated nodes

^2^ Secondary-calibrated node

## Discussion

### Substitution saturation

Results from saturation tests and plots suggest that phylogenetic signal in the sampled genes is unlikely to be erased or confounded by substitution saturation. While *nd2* third positions show some signs of saturation (at relatively high levels of divergence), removal of these sites resulted in a similarly resolved topology, differing only in the placement of clades already weakly supported and sustained by particularly short branches. If third positions of *nd2* were indeed fully saturated, the resultant phylogenetic noise would be expected to affect primarily resolution at deeper divergences. However, this was not the case, as most nodes inferred with putatively saturated sites were almost identically resolved and supported. Therefore, it appears that either the presumed multiple substitutions have not occurred or any saturation-driven noise has been swamped by the phylogenetic signal in the remaining data.

### Citharinoid phylogeny and taxonomic considerations

Although similar in many respects, the model-based and parsimony topologies inferred in this study exhibit some noteworthy differences (Figure 7). However, because of our preference for model-based phylogenetic inference methods over parsimony when dealing with molecular data, and the fact that both likelihood and Bayesian topologies were fully concordant, better resolved, and with higher support than the parsimony tree, the following discussion is based primarily on the likelihood topology. We note also that our model-based topologies are in strong accord with the morphology-based tree of Vari [26], and thereby require considerably less invocation of homoplasy to explain the evolution of morphological traits than would the parsimony topology.

Monophyly of the suborder Citharinoidei and the family Distichodontidae has not been questioned in most phylogenetic treatments of the Characiformes and the Ostariophysi [20,21,32]. However, a recent study focused on characid interrelationships by Oliveira et al. [62] recovered *Citharinus* nested within the Distichodontidae. Recognizing that this conclusion contradicted robust morphological and molecular evidence, Oliveira et al. stressed the need for further investigation of this finding with increased taxon and character sampling. The results presented here strongly support reciprocal monophyly between citharinids and distichodontids, and so provide additional evidence for the continued recognition of these taxa. The problematic finding of Oliveira et al. is most likely a result of limited sampling of citharinoids given the Neotropical focus of their study.

**Figure 9:**
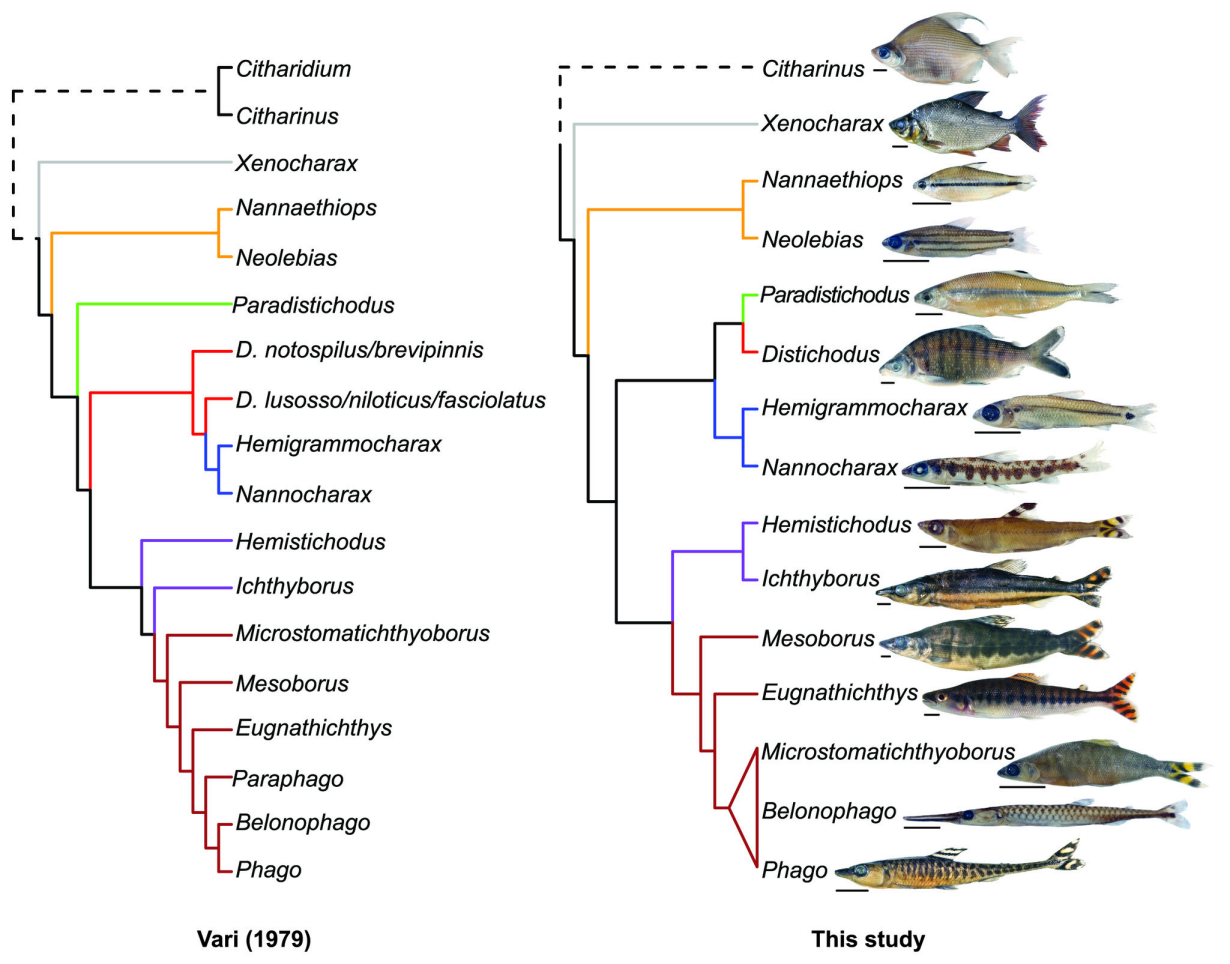
Comparison of citharinoid intergeneric relationships as inferred form anatomical [25] and molecular data (likelihood topology of this study).

In addition to corroborating distichodontid monophyly, our results provide strong support for the recognition of various suprageneric assemblages represented by well-supported clades (nodes B-G; Figures 4 and 5) and the intergeneric relationships entailed by the placement and composition of these clades. Results of the present study provide the first opportunity to test the hypothesis of distichodontid relationships arrived at by Vari [26] and, although derived from a different type of data and with only partially overlapping taxon sampling, the findings presented here are in general agreement with Vari’s morphology-based phylogeny, particularly with regard to the composition of the main suprageneric assemblages (Figure 9). It is worth noting that Vari’s study, being the first to apply cladistic methodology to the investigation of citharinoid relationships, predated the implementation of computer-assisted phylogenetic analyses. Therefore, no data matrix specifying character state distributions among sampled taxa was presented, and his resultant phylogeny is presumed to be derived from implementation of the “Hennigian Argumentation” procedure [4], which is not guaranteed to find the most parsimonious tree [99-101]. Notwithstanding, our results coincide with Vari’s in large degree (Figure 9), supporting: monophyly of both the Citharinoidei and the Distichodontidae (clade A), phylogenetic placement of *Xenocharax*, and a clade containing *Nannaethiops* and *Neolebias* (clade D), recognition of a clade containing *Nannocharax* and *Hemigrammocharax* (clade F), recognition of a clade containing *Hemistichodus, Ichthyborus*, *Microstomatichthyoborus, Mesoborus, Eugnatichthys, Phago* and *Belonophago* (clade J), and a subclade within that radiation containing *Microstomatichthyoborus, Mesoborus, Eugnatichthys, Phago* and *Belonophago* (clade G). Resolution within the abovementioned clades, however, differed between the two studies, and certain noteworthy novel relationships are suggested by our results. The overall congruence in the pattern of citharinoid relationships revealed by independent morphological and molecular datasets is notable and shows that, even in the absence of modern analytical methods, a detailed and thorough examination of morphology does quite well in providing a robust phylogenetic hypothesis.

*Xenocharax* is confirmed by our results as the sister to the remaining distichodontid radiation and, whereas currently considered monotypic [58], our study provide evidence in support for the validity of a second species, *X. crassus*, a Congo-Basin endemic that has long been considered a synonym of *X. spilurus*, the type species of the genus [102]. Anatomical examination of *X. crassus* specimens confirms this species to be morphologically distinct from *X. spilurus*. Therefore, *X. crassus* Pellegrin 1900 is recognized here as a valid species, pending a more detailed taxonomic treatment and formal resurrection.

Monophyly of a clade containing *Nannaethiops* and *Neolebias* (clade D) is strongly supported in this study. However, *Neolebias* is rendered paraphyletic by the placement of *Nannaethiops* species, a result that is equally strongly supported. This finding is not unanticipated given the comments of previous authors [26,103]. In light of our findings and the minimal anatomical evidence in support of the reciprocal monophyly between these genera [26], we concur with Géry and Zarske [104] in concluding that *Neolebias* should be considered a junior synonym of *Nannaethiops*.

Although placement of the genus *Paradistichodus* in our study is not strongly supported, model-based analyses converge on a finding of *Paradistichodus* as the sister group of *Distichodus*. This result conflicts with Vari’s phylogeny, in which *Paradistichodus* is recovered more basally (Figures 2 and 9) as a result of lacking a series of somewhat subjectively designated characters associated with increasingly kinetic oral jaws. While our molecular data do not provide sufficient evidence to allow for a conclusive assignment of *Paradistichodus*, reexamination of Vari’s morphological data indicates that character states in *Paradistichodus* require recoding in many instances. Moreover, our own exploratory anatomical survey suggests that the placement of *Paradistichodus* within a clade containing members of *Hemigrammocharax*, *Nannocharax* and *Distichodus* (clade H) has morphological support.

Monophyly of the clade of African darters (*Nannocharax* and *Hemigrammocharax*; clade F) is well supported both by our results and Vari’s. However, reciprocal monophyly between these two genera is strongly refuted by our study. While taxon sampling of *Hemigrammocharax* (only 3 of 9 species) and *Nannocharax* (only 12 of 25 species) was notably muted, the sampled *Hemigrammocharax* species were consistently nested within different subclades of *Nannocharax* (Figures 4-6). Vari [26] questioned the reciprocal monophyly between *Hemigrammocharax* and *Nannocharax* noting that a single character discriminates between them (the presence of an incomplete lateral line in *Hemigrammocharax* vs. complete in *Nannocharax*). Roberts [105] had previously suggested that the presence of an incomplete lateral line in *Hemigrammocharax* species was probably the result of multiple and independent reductions from the plesiomorphic condition (i.e., complete lateral line). However, Vari and Géry [106] and Vari and Ferraris [107] argued that differences in the extent of pored lateral line scales between *Nannocharax* and *Hemigrammocharax* may be the result of ontogenetic variation instead, especially since the hypothesized apomorphic lateral line reduction is incongruent with the distribution of other hypothesized apomorphic characters. This is in agreement with the findings of Coenen and Teugels [108], who showed that variation in lateral line length between some *Nannocharax* and *Hemigrammocharax* species exhibited a unimodal, instead of the expected bimodal distribution. This finding therefore contradicts the existence of the gap that purportedly distinguishes the two genera and further strengthens the idea that completeness of lateral line is not a character indicative of evolutionary relatedness.

Based on our results and the fact that currently only a single character of questionable diagnostic and phylogenetic value serves to distinguish *Nannocharax* from *Hemigrammocharax*, continued recognition of these genera is untenable and therefore, pending a more detailed revisional study of all valid species, should be synonymized.

A particularly significant finding of Vari’s study was the recognition of a large monophyletic subgrouping of distichodontids comprising the African darters (*Nannocharax* and *Hemigrammocharax*) and the pan-African genus *Distichodus* (Figure 2), which together constitute over 58% of distichodontid species diversity. Anatomical support for what Vari considered to be “a very distinctive unit within distichodontids” is compelling, and comprises nine osteological and myological synapomorphies, all considered trophic-related modifications facilitating a unique type of horizontal motion of the lower jaw [26]. Our model-based phylogenies also retrieved this clade, albeit with the inclusion of *Paradistichodus* and weak nodal support (clade H). As noted previously, the placement of *Paradistichodus* within this assemblage is supported by reexamination of Vari’s anatomical data.

Although Vari found compelling support for a clade containing *Hemigrammocharax*, *Nannocharax*, and *Distichodus*, he could locate no unambiguously synapomorphic characters uniting the five *Distichodus* species included in his study. In fact, he noted that aspects of neurocranial architecture in *Distichodus lusosso*, *D. niloticus* (=*D. nefasch*), and *D. fasciolatus* suggested a closer relationship of these species with African darters than with the other *Distichodus* species included in his study (i.e., *D. notospilus* and *D. brevipinnis*). This proposed phylogenetic pattern, if verified, would render *Distichodus* paraphyletic. Despite these observations, Vari refrained from making taxonomic or nomenclatural changes, urging instead for further study to determine the distribution of these and other derived characters among the numerous *Distichodus* species. Contrary to Vari’s finding, *Distichodus* (16 of 23 species) was resolved as monophyletic by our data regardless of optimality criterion, yet with only moderate nodal support. Interestingly however, both parsimony (Figure 6) and model-based phylogenies (Figures 4 and 5) partition our sampling of *Distichodus* into two well-supported subclades, with *D. lusosso* and *D. fasciolatus* located in one, and *D. notospilus* in the other. However, the relatively low support for a monophyletic *Distichodus* in our data clearly indicates that this large genus of commercially important distichodontids is in need of further study employing both morphological and additional molecular data.

Our study provides strong support for a sister-group relationship between two ecomorphologically derived genera: *Ichthyborus* and *Hemistichodus*. This result was found regardless of optimality criterion (node E; Figures 4-6), and with particularly strong support in the likelihood and Bayesian trees (bootstrap and posterior probability values of 100 and 1.0, respectively). While Vari found no anatomical evidence for a close relationship between these two taxa (to the exclusion of other members of clade J), the robustness of this sister-group relationship—as recovered by DNA sequence data—predicts that further anatomical scrutiny of these taxa may reveal previously unrecognized synapomorphies uniting the two.

A particularly unexpected finding of the present study is the apparent polyphyly of the morphologically distinctive genus *Phago*. However, we view this result with reservation, and underscore that the branches separating *P. boulengeri* from *P. intermedius* and linking them with allied genera are weakly supported, as are most branches defining intergeneric relationships within this suprageneric clade (node G; Figures 4 and 5). According to Vari [26], evidence for the monophyly of *Phago* consists of two uniquely derived characters, namely the presence of heavily ossified, thickened, vertically elongate scales, and anteroventrally-curved premaxillae overlapping the anterior ends of the dentaries. Our own examination of *Phago* voucher specimens confirms the species identity of the sampled taxa, supports Vari’s conclusions regarding the anatomical evidence for the monophyly of this distinctive taxon, as well as the extensive morphological support for its sister-group relationship with the equally distinctive *Belonophago*. Therefore, while our results provide strong support for Vari’s hypothesized clade consisting of the genera *Microstomatichthyoborus*, *Mesoborus*, *Eugnathichthys*, *Phago* and *Belonophago* (clade G), it appears that the phylogenetic signal in our data is unable to unambiguously resolve relationships within that assemblage.

### Robustness of the inferred node ages to changes in parameter priors and calibration strategies

#### Impact of the shape of calibration prior densities

Of the analyses conducted to assess the impact of changes in the shape of log-normally distributed priors on divergence-time estimates (Analyses 1-5; Tables 3 and 5), only Analysis 2 (i.e., applying a “young” prior to the age of the MRCA of *Distichodus*) resulted in considerably different (younger) node ages. This result may be indicative of an interaction between prior informativeness and calibration node age, given that the proposed calibration priors for the *Distichodus* node were comparatively more informative than the corresponding priors for the citharinoid node (Figure 3; a-c vs. e-g), and that “younger” priors at a given calibration node were likewise more informative than “older” ones (Figure 3; d and h). If such an interaction does indeed exist, then the younger the calibration node the stronger the impact of using overly informative priors. This observation is in agreement with claims by previous authors that overly informative priors, particularly for younger fossils, may lead to biased underestimates of divergence times [54,109]. Similarly, our results agree with the suggestion that calibrations at deeper nodes are the most effective for obtaining precise—although not necessarily accurate—node age estimates [110,111]. In contrast with the findings of other empirical studies [55-57], our results indicate that divergence-time estimates are robust to (reasonable) changes in the soft maximum constraint of the fossil calibrations used. Therefore, conclusions from previous empirical studies may not necessarily be applicable to all molecular dating analyses, and instead, the impact of hyperparameter choice on divergence time estimation may depend on specific characteristics of the data and of the taxonomic group. Where the data are relatively uninformative, the prior is therefore likely to exert a greater influence on the posterior distribution of divergence times [110].

#### Impact of calibration data type: primary vs. secondary calibrations

The analysis relying exclusively on primary (i.e., fossil-based) calibrations (Analysis 7; Tables 3 and 5) resulted in divergence-time estimates almost twice as young as in the control (i.e., Analysis 1). This result does not seem to be related to the adequacy (or lack thereof) of secondary calibrations (i.e., Near et al’s [27] dates), but rather to the impact of the absence of an explicit (i.e., user-specified) prior for the age of the root. More precisely, the exceptionally young divergence-time estimates in Analysis 7 are presumed to be an artifact of the markedly young root-age prior implied by the combined effects of the calibration priors on other internal nodes and the (Yule process) tree prior [110]. Conversely, the analysis relying exclusively on secondary calibrations (Analysis 8; Tables 3 and 5) resulted in the oldest estimated node ages. While 10-20% older than in most analyses, these estimates are nonetheless more consistent with the control than those from Analysis 7, suggesting that Near et al’s [27] dates may indeed be a reasonable alternative to calibrate actinopterygian molecular clocks in the absence of fossil evidence. The results of these analyses also provide further support to the notion that, by detecting rate variation across different levels of divergence, multiple calibrations improve the accuracy of divergence-time estimates [55,112]. Similarly, these results suggest that calibrating molecular clocks with deeper nodes, especially the root of the tree, produces more precise, and possibly more accurate, divergence-time estimates [110].

#### Impact of non-informative priors on the parameters of the clock, speciation, and substitution models

While most molecular-dating studies employing BEAST appear to use default priors for the parameters of the clock, speciation, and substitution models, we felt it important to explore the effects of using uniform priors instead. The fact that applying uniform priors on the parameters of the UCLN relaxed clock model, the Yule process, and the substitution models (Analyses 9-16; Tables 3 and 5) resulted in negligible differences in the estimated node ages, suggests that explicit knowledge about the parameters that describe these priors may not be necessary for arriving at reliable divergence-time estimates. Future molecular-dating studies, however, should assess the effect of using non-informative priors, for explicit knowledge about these processes is often lacking and the results presented here may be contingent to our dataset.

Given that at present there is no method for the objective formulation of priors that accurately summarize all the available evidence, our approach (i.e., to assess the sensitivity of divergence-time estimates to variations in parameter priors and calibration strategies) offers a reasonable strategy to account for some of the uncertainties inherent to Bayesian approaches for dating molecular phylogenies. The overall robustness of citharinoid divergence-time estimates thus provides an inferred evolutionary timescale with a stronger sense of confidence than otherwise would have been the case.

### Timescale of citharinoid diversification and implications for characiform biogeography

Despite advances in analytical methods of divergence time estimation using comparative DNA sequence data, and their increasingly widespread use in molecular phylogenetics, little is know about the tempo of characiform evolution beyond the information contained in its modest fossil record. As a result, most biogeographic hypotheses proposed to explain distribution patterns of extant characiform lineages have been framed in accordance with temporal information derived from fossil evidence, yet fossils only provide minimum age estimates.

Unraveling the biogeographic history characiforms has been a challenging task, not only because of the lack of a comprehensive time-scaled phylogeny of the order, but also because of the multiple instances of continentally disjunct sister-group relationships, and the phylogenetic uncertainty regarding some of these divergences [20-22,24,32]. Although a variety of biogeographic hypotheses have been proposed, most of which may be testable with divergence-time estimates from molecular phylogenies, there is currently no widely accepted explanation for the distribution of extant characiform lineages.

Perhaps the most popular of such hypotheses is the one that attributes the unbalanced Afro-Neotropical distribution of the order to a vicariance model coupled with extinction of numerous lineages in Africa [14]. Since any number of diversification scenarios invoking extinction may fit distribution patterns of extant taxa, testing the extinction component of this hypothesis is particularly problematic. Moreover, although in principle extinction may be verifiable (with fossils of extinct members), absence of evidence (i.e., fossils) does not imply evidence of absence [113]; extinction hypotheses are ultimately unfalsifiable. In any case, fossils assignable to Neotropical characiform lineages have not been found in Africa [23]. Lastly, this particular vicariance model implies a homogeneous distribution of early characiform lineages across western Gondwana before the break-up. Such a biogeographic pattern, however, is seldom observed in modern continental ichthyofaunas, which are instead generally unevenly distributed across regions and drainage basins [38,114]. A testable prediction of this hypothesis, however, is that the ancestral lineages leading to modern characiforms must have diversified well before the African/South American drift-vicariance event, and therefore, based on geological estimates for these palaeogeographic event [115,116], the divergence between citharinoids and the remaining members of the order (i.e., the MRCA of extant characiforms) must date to at least 100 Ma.

Other attempts at explaining transcontinental sister-group relationships among characiforms have resorted to more complex vicariance models, such as that proposed by Maisey [16]. This model, while not exclusively devised to address characiform biogeography, suggests that transatlantic disjunctions are most likely the result of multiple (instead of a single) vicariant episodes, including earlier tectonic events that may have driven pre-drift intercontinental divergences of Early Cretaceous freshwater fishes in western Gondwana. Testing this scenario, however, would require a comprehensive time-scaled phylogeny of the entire order (currently unavailable) so that the absolute ages of all transcontinental divergences and the timing and geometry of western Gondwana break-up models can be confronted and assessed for congruence.

Various authors have invoked marine dispersal to explain characiform distributions, arguing that it should not be excluded a priori when a simple model of vicariance does not readily explain present-day distributions [21,24,117,118]. Although biogeographic hypotheses involving dispersal are generally regarded as untestable, we agree that marine dispersal should not be invoked unless vicariance hypotheses have been already falsified.

In the first study applying molecular-dating techniques to investigate the timing of diversification in a clade of characiform fishes, Arroyave and Stiassny [24] presented a time-scaled molecular phylogeny of the African family Alestidae that also included (as outgroup taxa) representatives of the remaining African families and of various Neotropical lineages. Although their estimated mean ages for the MRCA of the Characiformes and the MRCA of the Citharinoidei (*ca* 87 and *ca* 67 Ma, respectively) might be problematic given their limited sampling of Neotropical characiforms and the fact that fossil calibrations were restricted to the alestid clade, these ages, even if only fairly accurate, imply that the origins of characiform fishes most likely postdate the mid-Cretaceous break-up of western Gondwana and their present-day distribution could not be attributed to any of the abovementioned vicariance hypotheses.

By contrast, the results presented here provide a considerably older age estimate for the origins of the citharinoid clade (*ca* 90 Ma; 95% HPD=110-73), which was the focus of our study. Assuming the mean age estimate derived here is a reasonable approximation of the actual divergence time between citharinids and distichodontids, it follows that the origins of the Characiformes would necessarily be considerably older than suggested by Arroyave and Stiassny [24], and as a result most likely older than the mid-Cretaceous break-up of Gondwana. Indeed, our results conform to available paleontological evidence, as the oldest characiform fossils date to the Cenomanian (*ca* 95 Ma) [23,119,120] and thus, assuming temporal gaps in the fossil record, the MRCA of characiforms must have already been present in the Early Cretaceous. Likewise, our results conform to the estimated age of the node representing the MRCA of the Characiformes (108 Ma; 95% HPD=135-79) by a recent study on the phylogenetic relationships and tempo of diversification of bony fishes [121].

Several authors have argued that divergence-time estimates become more accurate as the number of reliable calibrations increases [122,123], particularly for relaxed-clock methods where multiple calibrations act as landmark points detecting rate variation at different levels of divergence [112]. Therefore, given that Arroyave and Stiassny’s chronogram was based only on two fossil alestid (ingroup) calibrations [24], the estimated age for the origins of citharinoids presented here is deemed considerably more reliable. Additionally, the hypothesis of a Gondwanan origin for the Characiformes is further supported by the fact that our divergence-time estimates are robust to changes in calibration and analysis settings.

By reconciling molecular-clock and fossil-based estimates of clade ages, our results have noteworthy implications for understanding characiform historical biogeography. Namely, they provide independent temporal evidence in support for the hypothesis that attributes the disjunct distribution between African citharinoids and Neotropical characiforms to the mid-Cretaceous fragmentation of western Gondwana. Likewise, if the timing of divergence of African alestids and hepsetids from their respective Neotropical sister groups is correspondingly older than proposed by Arroyave and Stiassny [24], then explaining the modern distribution of Characiformes would not necessitate invocation of post-drift dispersal [21,124], but simply an African/South American drift-vicariance event coupled with differential distribution patterns of primeval characiform lineages inhabiting Gondwana before the break-up [23].

While it is expected for divergence-time estimates based on log-normally distributed priors to be older than calibration fossils, our inferred node age for the MRCA of Citharinoidei is substantially older than its corresponding calibration fossil (Figure 8). As recently noted by Near et al. [27], explaining such large discrepancies requires invoking temporally large gaps in the fossil record. Interestingly, except for a few isolated teeth from the Late Cretaceous/Early Paleocene, there is indeed a relatively large gap (~40 Ma; Ypresian-Cenomanian) in the stratigraphic distribution of characiform fossils [23] that broadly corresponds to the difference between the molecular clock- and fossil-based citharinoid clade age estimates. While the taxonomic structure of the fossil record is largely shaped by sampling bias [125], such a gap in the stratigraphic distribution of characiform fossils might be real (either because of low levels of diversity in the early stages of characiform evolution and/or because of lack of suitable fossil deposits). Regardless, future paleontological research should be aimed at filling the gaps in the still meager characiform fossil record, since additional fossils would necessarily lead to more accurate and reliable molecular-dated phylogenies.

While the estimated mean age for the origin of the Distichodontidae proposed here (*ca* 67 Ma) is older than that estimated for the Alestidae (*ca* 54 Ma) [24], it is noteworthy that for both families the origins of modern genera (and most cladogenetic events) appear to have occurred between the Early Oligocene and the Late Miocene, during nearly the same time interval (*ca* 30–10 Ma). The lower bound of this interval coincides with the Eocene-Oligocene tectonic uplift of eastern Africa (*ca* 40-30 Ma), a geologic event that profoundly affected the geometry of contemporary African rivers [126,127] and initiated the development of the modern Congo Basin [128-130]. The striking overlap in the current distribution patterns of alestids and distichodontids, both with highest species richness and endemism concentrated in the Congo Basin, coupled with the idea that diversification in these families was broadly concurrent with the early stages of development of the modern drainage of the Congo River, suggests that diversification and biogeographic patterns in alestids and distichodontids—and possibly numerous other groups of African freshwater fishes—may have been greatly influenced by the Neogene reconfiguration of drainage patterns in Central Africa.

We acknowledge that based on our results it is difficult to assume, let alone confirm, a causal relationship between the geotectonic events that shaped the modern Central African drainage system and the diversification of alestids and distichodontids. Nevertheless, our discovery of spatio-temporal congruencies between cladogenetic and palaeohydrologic events conforms to a growing body of evidence indicating that diversification in African freshwater fishes was profoundly influenced by Oligocene and Miocene tectonism [25,34,131]. Future research aimed at detecting temporal shifts in diversification rates and phylogeographic signatures of drainage evolution may provide additional evidence to further test the influence of palaeogeographic processes (e.g., drainage basin isolation and recapture) on the evolution of these clades.

## Supporting Information

Figure S1 **Saturation plots.** Scatterplots of observed number of transitions and transversions against corrected genetic distance for third codon positions of each gene sampled in this study. X-axis corresponds to observed transitions (s) and transversions (v) and Y-axis corresponds to corrected genetic distances (d) based on best-fit substitution models.
(TIF)

Table S1 **Taxon and character sampling.** Taxa, voucher catalog numbers, and GenBank accession numbers for the gene sequences included in the analyses. Institutional abbreviations: AMNH (American Museum of Natural History), CU (Cornell University Museum of Vertebrates).
(DOCX)
