# Supplementary figures and images for: Are characiform Fishes Gondwanan in Origin? Insights from a Time-Scaled Molecular Phylogeny of the Citharinoidei (Ostariophysi: Characiformes)
Source: PLoS One. 2013 Oct 8;8(10):e77269. doi: 10.1371/journal.pone.0077269 (PMC3792904; doi:10.1371/journal.pone.0077269)

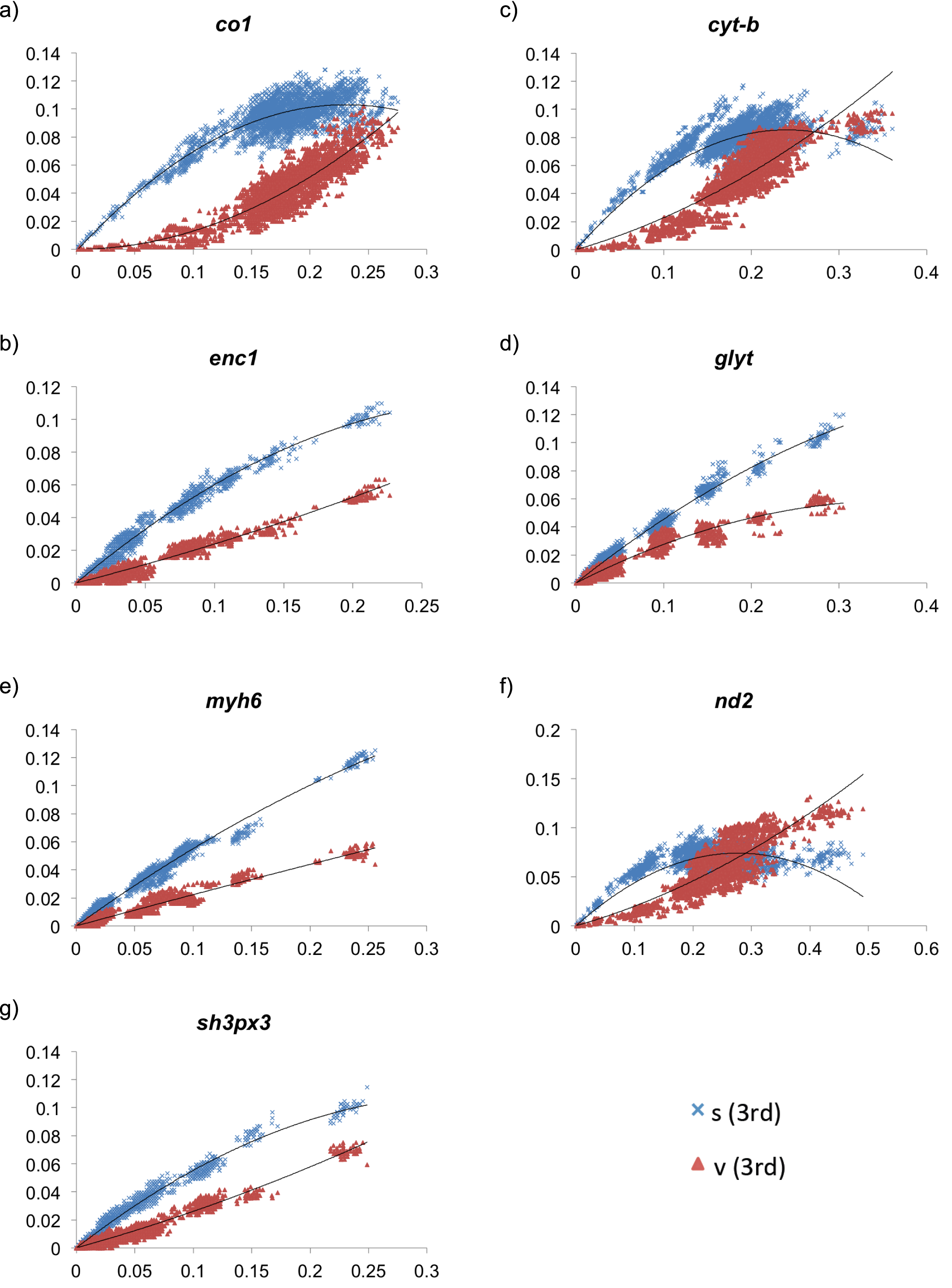

Supplement: Figure S1 — Saturation plots. Scatterplots of observed number of transitions and transversions against corrected genetic distance for third codon positions of each gene sampled in this study. X-axis corresponds to observed transitions (s) and transversions (v) and Y-axis corresponds to corrected genetic distances (d) based on best-fit substitution models. (TIF) [file pone.0077269.s001.tif]
