# Supplementary material for: Are characiform Fishes Gondwanan in Origin? Insights from a Time-Scaled Molecular Phylogeny of the Citharinoidei (Ostariophysi: Characiformes)
Source: PLoS One. 2013 Oct 8;8(10):e77269. doi: 10.1371/journal.pone.0077269 (PMC3792904; doi:10.1371/journal.pone.0077269)
Supplement: Table S1 — Taxon and character sampling. Taxa, voucher catalog numbers, and GenBank accession numbers for the gene sequences included in the analyses. Institutional abbreviations: AMNH (American Museum of Natural History), CU (Cornell University Museum of Vertebrates). (DOCX) [file pone.0077269.s002.docx]

**Table S1. Taxa, voucher catalog numbers^*^, and GenBank accession numbers for the gene sequences included in the analyses.**

| **Taxon** | **Voucher** | **GenBank Accession Number** | | | | | | |
| --- | --- | --- | --- | --- | --- | --- | --- | --- |
|  |  | ***co1*** | ***cyt-b*** | ***enc1*** | ***glyt*** | ***myh6*** | ***nd2*** | ***sh3px3*** |
| OUTGROUP |  |  |  |  |  |  |  |  |
| Cyprinidae |  |  |  |  |  |  |  |  |
| *Danio rerio* | n/a | NC_002333 | NC_002333 | EF032975 | EF032988 | EF032923 | NC_002333 | EF033001 |
| Ictaluridae |  |  |  |  |  |  |  |  |
| *Ictalurus punctatus* | n/a | AF482987 | AF482987 | EF032981 | EF032994 | EF032929 | AF482987 | EF033007 |
| INGROUP |  |  |  |  |  |  |  |  |
| Citharinidae |  |  |  |  |  |  |  |  |
| *Citharinus citharus* | AMNH 226441 | KF541749 | KF541863 | KF541974 | KF542085 | KF542201 | KF542320 | KF542432 |
| *Citharinus citharus* | AMNH 226441 | KF541750 | KF541864 | KF541975 | KF542086 | KF542202 | KF542321 | KF542433 |
| *Citharinus congicus* | AMNH 252692 | KF541752 | KF541866 | KF541977 | KF542090 | KF542209 | KF542323 | KF542435 |
| *Citharinus congicus* | AMNH 240019 | KF541751 | KF541865 | KF541976 | KF542089 | KF542208 | KF542322 | KF542434 |
| *Citharinus gibbosus* | AMNH 240020 | KF541754 | KF541868 | KF541979 | KF542092 | KF542204 | KF542325 | KF542437 |
| *Citharinus gibbosus* | AMNH 243512 | KF541755 | KF541871 | KF541980 | KF542093 | KF542205 | KF542326 | KF542440 |
| *Citharinus gibbosus* | AMNH 238209 | KF541753 | KF541867 | KF541978 | KF542091 | KF542203 | KF542324 | KF542436 |
| *Citharinus sp.* | CU 92980 | KF541756 | KF541869 | KF541981 | KF542087 | KF542206 | KF542327 | KF542438 |
| *Citharinus sp.* | CU 92980 | KF541757 | KF541870 | KF541982 | KF542088 | KF542207 | KF542328 | KF542439 |
| Distichodontidae |  |  |  |  |  |  |  |  |
| *Belonophago hutsebouti* | AMNH 241850 | KF541759 | KF541872 | KF541983 | KF542094 | KF542210 | KF542329 | KF542441 |
| *Belonophago hutsebouti* | AMNH 241851 | KF541760 | KF541873 | KF541984 | KF542095 | KF542211 | KF542330 | KF542442 |
| *Belonophago tinanti* | AMNH 238286 | KF541758 | KF541874 | KF541985 | KF542096 | KF542212 | KF542331 | KF542443 |
| *Distichodus affinis* | AMNH 252633 | KF541762 | KF541891 | KF541997 | KF542130 | KF542213 | KF542343 | KF542538 |
| *Distichodus affinis* | AMNH 247062 | KF541761 | KF541890 | KF541996 | KF542110 | KF542236 | KF542342 | KF542537 |
| *Distichodus antonii* | CU 95832 | KF541764 | KF541900 | KF541999 | KF542118 | KF542214 | KF542355 | KF542539 |
| *Distichodus antonii* | AMNH 246450 | KF541763 | KF541899 | KF541998 | KF542117 | KF542272 | KF542354 | KF542444 |
| *Distichodus atroventralis* | AMNH 246956 | KF541765 | KF541903 | KF542000 | KF542119 | KF542237 | KF542356 | KF542445 |
| *Distichodus atroventralis* | AMNH 255281 | KF541766 | KF541904 | KF542001 | KF542122 | KF542238 | KF542357 | KF542446 |
| *Distichodus decemmaculatus* | AMNH 247931 | KF541767 | KF541920 | KF542002 | KF542112 | KF542246 | KF542344 | KF542447 |
| *Distichodus decemmaculatus* | AMNH 252263 | KF541768 | KF541921 | KF542003 | KF542111 | KF542247 | KF542345 | KF542540 |
| *Distichodus engycephalus* | CU 94663 | KF541798 | KF541895 | KF542006 | KF542133 | KF542275 | KF542370 | KF542450 |
| *Distichodus engycephalus* | AMNH 257169 | KF541796 | KF541893 | KF542004 | KF542131 | KF542273 | KF542368 | KF542448 |
| *Distichodus engycephalus* | AMNH 257704 | KF541797 | KF541894 | KF542005 | KF542132 | KF542274 | KF542369 | KF542449 |
| *Distichodus fasciolatus* | AMNH 240040 | KF541799 | KF541909 | KF542007 | KF542126 | KF542239 | KF542359 | KF542451 |
| *Distichodus fasciolatus* | AMNH 253304 | KF541800 | KF541910 | KF542008 | KF542120 | KF542240 | KF542360 | KF542452 |
| *Distichodus hypostomatus* | AMNH 249522 | KF541834 | KF541918 | KF542009 | KF542115 | KF542300 | KF542372 | KF542453 |
| *Distichodus hypostomatus* | AMNH 249522 | KF541835 | KF541919 | KF542010 | KF542116 | KF542301 | KF542373 | KF542454 |
| *Distichodus kolleri* | CU 93515 | KF541802 | KF541912 | KF542012 | KF542135 | KF542249 | KF542351 | KF542456 |
| *Distichodus kolleri* | AMNH 236538 | KF541801 | KF541911 | KF542011 | KF542134 | KF542248 | KF542350 | KF542455 |
| *Distichodus lusosso* | AMNH 247230 | KF541803 | KF541901 | KF542013 | KF542121 | KF542215 | KF542365 | KF542457 |
| *Distichodus lusosso* | AMNH 250310 | KF541804 | KF541902 | KF542014 | KF542125 | KF542216 | KF542366 | KF542458 |
| *Distichodus maculatus* | CU 91523 | KF541805 | KF541896 | KF542015 | KF542127 | KF542276 | KF542364 | KF542459 |
| *Distichodus maculatus* | CU 95265 | KF541769 | KF541892 | KF542016 | KF542128 | KF542277 | KF542371 | KF542460 |
| *Distichodus mossambicus* | AMNH 251295 | KF541806 | KF541907 | KF542017 | KF542123 | KF542241 | KF542363 | KF542461 |
| *Distichodus mossambicus* | AMNH 253393 | KF541807 | KF541908 | KF542018 | KF542124 | KF542242 | KF542358 | KF542462 |
| *Distichodus noboli* | AMNH C07-560 | KF541771 | KF541923 | KF542020 | KF542138 | KF542261 | KF542349 | KF542464 |
| *Distichodus noboli* | AMNH 247930 | KF541770 | KF541922 | KF542019 | KF542137 | KF542260 | KF542348 | KF542463 |
| *Distichodus notospilus* | AMNH 249523 | KF541772 | KF541898 | KF542066 | KF542141 | KF542298 | KF542352 | KF542465 |
| *Distichodus notospilus* | AMNH 249537 | KF541773 | KF541897 | KF542067 | KF542136 | KF542299 | KF542353 | KF542466 |
| *Distichodus petersii* | CU 93783 | KF541774 | KF541913 | KF542057 | KF542129 | KF542262 | KF542367 | KF542541 |
| *Distichodus sexfasciatus* | AMNH 251287 | KF541776 | KF541906 | KF542058 | KF542140 | KF542244 | KF542362 | KF542468 |
| *Distichodus sexfasciatus* | AMNH 240874 | KF541775 | KF541905 | KF542021 | KF542139 | KF542243 | KF542361 | KF542467 |
| *Distichodus teugelsi* | AMNH 253625 | KF541777 | KF541924 | KF542022 | KF542113 | KF542250 | KF542346 | KF542469 |
| *Distichodus teugelsi* | AMNH 253758 | KF541778 | KF541925 | KF542046 | KF542114 | KF542251 | KF542347 | KF542470 |
| *Eugnathichthys macroterolepis* | AMNH 253084 | KF541780 | KF541876 | KF541987 | KF542098 | KF542218 | KF542333 | KF542503 |
| *Eugnathichthys macroterolepis* | AMNH 245508 | KF541779 | KF541875 | KF541986 | KF542097 | KF542217 | KF542332 | KF542502 |
| *Eugnathichthys sp.nov.* | AMNH 249790 | KF541782 | KF541878 | KF541989 | KF542100 | KF542292 | KF542341 | KF542505 |
| *Eugnathichthys sp.nov.* | AMNH 246319 | KF541781 | KF541877 | KF541988 | KF542099 | KF542291 | KF542340 | KF542504 |
| *Hemigrammocharax multifasciatus* | CU 91291 | KF541808 | KF541939 | KF542023 | KF542154 | KF542293 | KF542387 | KF542527 |
| *Hemigrammocharax ocellicauda* | AMNH 241885 | KF541809 | KF541955 | KF542024 | KF542174 | KF542219 | n/a | KF542513 |
| *Hemigrammocharax ocellicauda* | AMNH 247924 | KF541811 | KF541957 | KF542026 | KF542176 | KF542221 | n/a | KF542472 |
| *Hemigrammocharax ocellicauda* | AMNH 241885 | KF541810 | KF541956 | KF542025 | KF542175 | KF542220 | n/a | KF542471 |
| *Hemigrammocharax uniocellatus* | AMNH 252623 | n/a | KF541953 | KF542027 | KF542155 | KF542278 | KF542388 | KF542473 |
| *Hemigrammocharax uniocellatus* | AMNH 252623 | n/a | KF541954 | KF542028 | KF542156 | KF542279 | KF542389 | KF542474 |
| *Hemistichodus lootensi* | AMNH 242509 | KF541845 | KF541959 | KF542030 | KF542143 | KF542223 | KF542405 | KF542476 |
| *Hemistichodus lootensi* | AMNH 242506 | KF541844 | KF541958 | KF542029 | KF542142 | KF542222 | KF542404 | KF542475 |
| *Hemistichodus mesmaerksi* | AMNH 252258 | KF541847 | KF541961 | KF542032 | KF542145 | KF542309 | KF542407 | KF542478 |
| *Hemistichodus mesmaerksi* | AMNH 242505 | KF541846 | KF541960 | KF542031 | KF542144 | KF542308 | KF542406 | KF542477 |
| *Ichthyborus besse* | CU 94491 | KF541843 | KF541970 | KF542081 | KF542150 | KF542280 | KF542394 | KF542515 |
| *Ichthyborus ornatus* | CU 92917 | KF541838 | KF541973 | KF542084 | KF542179 | KF542283 | KF542397 | KF542481 |
| *Ichthyborus ornatus* | AMNH 249768 | KF541837 | KF541972 | KF542083 | KF542178 | KF542282 | KF542396 | KF542480 |
| *Ichthyborus ornatus* | AMNH 238282 | KF541836 | KF541971 | KF542082 | KF542151 | KF542281 | KF542395 | KF542479 |
| *Ichthyborus quadrilineatus* | AMNH 257081 | KF541839 | n/a | KF542033 | KF542152 | KF542289 | KF542398 | KF542516 |
| *Ichthyborus quadrilineatus* | AMNH 257096 | KF541840 | n/a | KF542034 | KF542153 | KF542290 | KF542399 | KF542517 |
| *Mesoborus crocodilus* | AMNH 252426 | KF541784 | KF541881 | KF541991 | KF542105 | KF542225 | KF542335 | KF542533 |
| *Mesoborus crocodilus* | CU 91474 | KF541785 | KF541880 | KF541992 | KF542106 | KF542226 | KF542336 | KF542534 |
| *Mesoborus crocodilus* | AMNH 249770 | KF541783 | KF541879 | KF541990 | KF542104 | KF542224 | KF542334 | KF542482 |
| *Microstomatichthyoborus bashforddeani* | CU 91515 | KF541788 | KF541889 | KF541995 | KF542109 | KF542229 | KF542386 | KF542508 |
| *Microstomatichthyoborus bashforddeani* | AMNH 257161 | KF541786 | KF541887 | KF541993 | KF542107 | KF542227 | KF542384 | KF542506 |
| *Microstomatichthyoborus bashforddeani* | AMNH 257162 | KF541787 | KF541888 | KF541994 | KF542108 | KF542228 | KF542385 | KF542507 |
| *Nannaethiops bleheri* | CU 94489 | KF541848 | KF541926 | KF542047 | KF542185 | KF542315 | KF542374 | KF542518 |
| *Nannaethiops unitaeniatus* | AMNH 249555 | KF541849 | KF541928 | KF542048 | KF542186 | KF542316 | KF542375 | KF542519 |
| *Nannaethiops unitaeniatus* | AMNH 249555 | KF541850 | KF541929 | KF542049 | KF542187 | KF542317 | KF542376 | KF542536 |
| *Nannocharax altus* | AMNH 253852 | KF541812 | n/a | KF542050 | KF542169 | KF542284 | KF542424 | KF542483 |
| *Nannocharax altus* | AMNH 253991 | KF541829 | n/a | KF542051 | KF542172 | KF542287 | KF542427 | KF542485 |
| *Nannocharax altus* | AMNH 253854 | KF541813 | n/a | KF542053 | KF542170 | KF542285 | KF542425 | KF542484 |
| *Nannocharax altus* | AMNH 253854 | KF541814 | n/a | KF542054 | KF542171 | KF542286 | KF542426 | KF542514 |
| *Nannocharax ansorgii* | AMNH 257013 | KF541815 | KF541951 | KF542063 | KF542157 | KF542230 | KF542408 | KF542486 |
| *Nannocharax ansorgii* | AMNH 257013 | KF541816 | KF541952 | KF542064 | KF542158 | KF542231 | KF542409 | KF542487 |
| *Nannocharax brevis* | AMNH 253723 | KF541817 | KF541962 | KF542035 | KF542177 | KF542245 | KF542429 | KF542488 |
| *Nannocharax elongatus* | AMNH 251111 | KF541819 | KF541941 | KF542061 | KF542160 | KF542253 | KF542414 | KF542490 |
| *Nannocharax elongatus* | AMNH 242261 | KF541818 | KF541940 | KF542060 | KF542159 | KF542252 | KF542413 | KF542489 |
| *Nannocharax fasciatus* | AMNH 254206 | KF541820 | n/a | KF542036 | KF542164 | KF542307 | KF542418 | n/a |
| *Nannocharax fasciatus* | AMNH 257016 | KF541841 | KF541942 | n/a | KF542180 | KF542270 | KF542419 | KF542528 |
| *Nannocharax fasciatus* | AMNH 257016 | KF541842 | KF541943 | n/a | KF542181 | KF542271 | KF542420 | KF542529 |
| *Nannocharax hollyi* | CU 91674 | KF541821 | n/a | KF542062 | KF542165 | KF542254 | KF542430 | KF542535 |
| *Nannocharax occidentalis* | AMNH 256973 | KF541822 | KF541944 | KF542037 | KF542182 | KF542232 | KF542421 | KF542530 |
| *Nannocharax occidentalis* | AMNH 256973 | KF541823 | KF541945 | KF542065 | KF542183 | KF542255 | KF542422 | KF542531 |
| *Nannocharax parvus* | AMNH 253853 | KF541824 | KF541947 | KF542055 | KF542173 | KF542288 | KF542428 | KF542491 |
| *Nannocharax procatopus* | AMNH 251110 | KF541826 | KF541949 | n/a | KF542162 | KF542264 | KF542416 | KF542493 |
| *Nannocharax procatopus* | AMNH 251110 | KF541827 | KF541950 | n/a | KF542163 | KF542265 | KF542417 | KF542494 |
| *Nannocharax procatopus* | AMNH 243636 | KF541825 | KF541948 | n/a | KF542161 | KF542263 | KF542415 | KF542492 |
| *Nannocharax rubrolabiatus* | CU 93458 | n/a | KF541946 | KF542059 | KF542184 | KF542256 | KF542423 | KF542532 |
| *Nannocharax schoutedeni* | AMNH 241889 | KF541861 | KF541963 | KF542038 | KF542166 | KF542302 | n/a | KF542495 |
| *Nannocharax schoutedeni* | AMNH 241890 | KF541862 | KF541964 | KF542039 | KF542167 | KF542303 | n/a | KF542496 |
| *Nannocharax sp. "So'o"* | AMNH 241774 | KF541828 | KF541965 | KF542056 | KF542168 | KF542259 | KF542431 | n/a |
| *Neolebias ansorgii* | CU 93512 | KF541860 | KF541938 | KF542080 | KF542196 | KF542295 | KF542412 | KF542548 |
| *Neolebias ansorgii* | AMNH 250442 | KF541858 | KF541936 | KF542078 | KF542194 | KF542306 | KF542410 | KF542546 |
| *Neolebias ansorgii* | AMNH 251003 | KF541859 | KF541937 | KF542079 | KF542195 | KF542294 | KF542411 | KF542547 |
| *Neolebias gracilis* | AMNH 241888 | KF541851 | KF541930 | KF542073 | KF542188 | KF542310 | KF542377 | KF542520 |
| *Neolebias gracilis* | AMNH 241888 | KF541852 | KF541931 | KF542074 | KF542189 | KF542311 | KF542378 | KF542521 |
| *Neolebias trewavasae* | AMNH 249536 | KF541853 | KF541927 | KF542052 | KF542193 | KF542318 | KF542382 | KF542525 |
| *Neolebias trewavasae* | AMNH uncataloged | KF541857 | KF541935 | n/a | n/a | KF542319 | KF542383 | KF542526 |
| *Neolebias trilineatus* | CU 91513 | KF541856 | KF541934 | KF542077 | KF542192 | KF542314 | KF542381 | KF542524 |
| *Neolebias trilineatus* | AMNH 247928 | KF541854 | KF541932 | KF542075 | KF542190 | KF542312 | KF542379 | KF542522 |
| *Neolebias trilineatus* | AMNH 247928 | KF541855 | KF541933 | KF542076 | KF542191 | KF542313 | KF542380 | KF542523 |
| *Paradistichodus dimidiatus* | AMNH 257747 | KF541830 | KF541914 | KF542040 | KF542146 | KF542266 | KF542390 | KF542497 |
| *Paradistichodus dimidiatus* | AMNH 257747 | KF541831 | KF541915 | KF542041 | KF542147 | KF542267 | KF542391 | KF542498 |
| *Paradistichodus dimidiatus* | AMNH 257765 | KF541832 | KF541916 | KF542042 | KF542148 | KF542268 | KF542392 | KF542499 |
| *Paradistichodus dimidiatus* | AMNH 257765 | KF541833 | KF541917 | KF542043 | KF542149 | KF542269 | KF542393 | KF542500 |
| *Phago boulengeri* | CU 91510 | KF541791 | KF541884 | KF542072 | KF542103 | KF542233 | KF542339 | KF542510 |
| *Phago boulengeri* | AMNH 240067 | KF541789 | KF541882 | KF542070 | KF542101 | KF542257 | KF542337 | KF542501 |
| *Phago boulengeri* | AMNH 241663 | KF541790 | KF541883 | KF542071 | KF542102 | KF542258 | KF542338 | KF542509 |
| *Phago intermedius* | AMNH 252519 | KF541792 | KF541885 | n/a | n/a | KF542234 | n/a | KF542511 |
| *Phago intermedius* | CU 92907 | KF541793 | KF541886 | n/a | n/a | KF542235 | n/a | KF542512 |
| *Xenocharax crassus* | AMNH 249785 | KF541794 | KF541966 | KF542068 | KF542197 | KF542296 | KF542400 | KF542542 |
| *Xenocharax crassus* | CU 92908 | KF541795 | KF541967 | KF542069 | KF542198 | KF542297 | KF542401 | KF542543 |
| *Xenocharax spilurus* | AMNH 253876 | n/a | KF541968 | KF542044 | KF542199 | KF542304 | KF542402 | KF542544 |
| *Xenocharax spilurus* | AMNH 253910 | n/a | KF541969 | KF542045 | KF542200 | KF542305 | KF542403 | KF542545 |

*Institutional abbreviations: AMNH (American Museum of Natural History), CU (Cornell University Museum of Vertebrates).
